# Video-based interventions to promote HPV vaccination among individuals aged 9 to 26: a systematic review and meta-analysis

**DOI:** 10.1186/s12889-026-26759-w

**Published:** 2026-03-14

**Authors:** ChengChing Liu, Teresa J. Ng, Sudaba Mansuri, Nouran Ghonaim, Dibakar Roy, Umniah Moshi, Angela Chia-Chen Chen

**Affiliations:** 1 College of Nursing, Michigan State University, East Lansing, MI 48864 USA; 2 Lyman Briggs College, Michigan State University, East Lansing, MI 48864 USA; 3 College of Natural Science, Michigan State University, East Lansing, MI 48864 USA

**Keywords:** Human papillomavirus (HPV), HPV vaccine, Video intervention, Systematic review and meta-analysis, 9–26 years old

## Abstract

Human papillomavirus (HPV) is the most prevalent sexually transmitted infection in the U.S. and is associated with approximately 40,000 new cancer cases each year, affecting both males (e.g., oropharyngeal, anal cancers) and females (e.g., cervical, vaginal cancers). The CDC recommends HPV vaccination for individuals aged 9 to 26, and studies have shown that HPV vaccines are effective in reducing the rates of cancers. Video-based interventions may be an effective strategy to increase vaccination uptake. This study aimed to systematically review the literature on using video interventions to promote HPV vaccination outcomes (knowledge, attitudes, beliefs, self-efficacy, and uptake). A systematic review protocol was developed following PRISMA-P guidelines. Databases searched included CINAHL, PubMed, ERIC, and PsycINFO, using keywords from a comprehensive review of HPV vaccination interventions and videos, with no date restrictions. Inclusion criteria focused on RCTs and quasi-experimental studies using videos as interventions. Titles, abstracts, and full texts were screened independently by two reviewers following PRISMA guidelines. Pooled effect sizes (Hedge’s *g)* were calculated using random effects modeling via the Comprehensive Meta-Analysis software. Eighteen articles, including 5,873 participants (ranging from 35 to 739), were included in this review. The majority of these studies were conducted in the U.S. (k = 12, 66.6%), RCTs (k = 7, 38.8%), and included females (k = 16, *n* = 4,956, 84.4%). Most studies employed informational videos (*n* = 11, 61.2%), while five used narrative/storytelling videos. Four studies that combined videos with additional strategies. Of the studies reporting HPV vaccine outcomes (k = 8), initiation rates ranged from 2.4% to 51.9%. All eight studies (100%) reported significant improvements in HPV, vaccine, and/or cervical cancer knowledge. The five studies examining vaccine intention showed consistent increases in intent to vaccinate. Nine studies were included in the meta-analysis, yielding pooled effect sizes ranging from small to moderate (*g* = 0.16 to 0.62)*,* indicating a modest but meaningful impact of video-based interventions on HPV-related outcomes. This review underscores the need to expand video-based interventions to promote HPV vaccination beyond traditional methods like education and flyers. More focus is needed on males aged 9 to 26, as most studies targeted females. Developing and evaluating strategies, including social media interventions, could enhance HPV vaccine uptake among adolescents and young adults. PROSPERO ID: CRD42025650071.

**Trial registration**

PROSPERO ID: CRD42025650071.

**Supplementary Information:**

The online version contains supplementary material available at 10.1186/s12889-026-26759-w.

## Introduction

Human papillomavirus (HPV) is the most prevalent sexually transmitted infection worldwide [1]. It is responsible for 570,000 new cancer cases annually in females and 60,000 in males, making it a significant global public health concern [2]. Furthermore, there has been a notable rise in the rate of HPV diagnoses among men recently, and this trend is expected to surpass the infection rate in women eventually [3]. In addition to the increased HPV infection rate, 10–20% can persist and progress to various types of cancer. Persistent HPV infection is responsible for approximately 90% of anal and cervical cancers, 70% of vaginal and vulvar cancers, 60% of penile cancers, and up to 70% of oropharyngeal cancers [4, 5]. HPV-related cancers account for over 7,000 deaths annually, in addition to an estimated economic burden of $774 million USD [6]. Nearly 50% of the 14 million new HPV infections each year occur in individuals aged 15 to 24, highlighting the critical need for effective prevention strategies targeting younger populations to reduce the risk of both [7]. Additionally, the Centers for Disease Control and Prevention (CDC) reports that nearly 85% of people will be infected with HPV at some point in their lives [1]. According to the World Health Organization, one of its goals is to eliminate HPV-related cancers, particularly cervical cancer, worldwide by 2030 [8].

The HPV vaccine can prevent up to 90% of cancers caused by HPV and is recommended for individuals between 9 and 26 years of age. Its preventive impact is greatest when given between ages 9 and 14, as younger recipients mount a stronger immune response and are less likely to have been exposed to the virus through sexual activity [8–12]. Despite this, vaccination rates remain low. In the U.S., about 60% of adolescents were up-to-date on HPV vaccination in 2020, with 61% of females being fully vaccinated versus 56% of males [13]. The HPV vaccine is routinely recommended as a catch-up vaccination for adults aged 18–26 who were not adequately vaccinated earlier [14]. However, a 2022 national survey found that only 47% of surveyed college students (37% of men vs. 53% of women) had received one or more doses, falling short of the national goal of 80% [15, 16]. This underscores the need for more effective interventions to address this gap.

Digital and social media interventions have been applied to promote the HPV vaccination rate. For example, digital interventions can promote HPV vaccination by sending reminders to clients and providers and addressing barriers such as undervaluation, misconceptions, attitudinal and structural challenges, scheduling difficulties, and high costs, often delivered through mobile messaging, apps, websites, and social media [17]. However, a systematic review and meta-analysis of 34 studies found that the effectiveness of digital interventions varies by participant gender and platform, and it focused on interventions limited to text messages, emails, webinars, and website information, without targeting the CDC-recommended age group of 9 to 26 [17]. Another integrative review examined social media–assisted interventions, defined as those delivered through or facilitated by social media platforms. The review included 24 studies published between 2015 and 2021 and found that social media messages had a positive effect on improving HPV-related awareness and knowledge [18]. Only 10 of the 24 studies assessed vaccination initiation and completion rates, and none reported statistically significant effects [18]. Although most studies targeted adolescents, high school, and college students, participants reported paying little attention to sponsored health advertisements on social media. Additionally, HPV vaccination was often perceived as being associated with sexual behavior, highlighting the need to integrate emerging social media formats—such as short-form videos and interactive content—to increase engagement [18].

Evidence indicates that video-based interventions are effective in promoting vaccination intentions and uptake among youth, including college students. Narrative or storytelling interventions, in particular, offer a culturally relevant approach to fostering positive behavior change [19]. In one randomized controlled trial (RCT), young adults aged 18–26 who viewed narrative videos reported significantly higher vaccination intentions compared to those who received an educational pamphlet; however, vaccination status was not assessed [19]. Another RCT found that participants exposed to either a written pamphlet or a video about HPV and vaccination demonstrated greater knowledge and stronger vaccination intentions than those who received general cancer prevention materials, though follow-up data on vaccination behavior were lacking. Despite evidence supporting video-based interventions for improving HPV vaccination outcomes, uncertainty remains regarding which formats are most effective and how effectiveness varies across study and population characteristics.

In this study, the term “video-based” refers to interventions or educational materials that use video as the primary medium for delivering content, including narrative, animated, and documentary-style formats—all shown to influence health knowledge and behavior. Although social media and virtual reality formats were not explicitly targeted, studies employing these platforms were included when structured video content served as the main educational component. This systematic review and meta-analysis contributes to the existing literature by specifically examining the effectiveness of video- and narrative-based digital interventions for HPV vaccination, highlighting their potential to engage younger populations, such as college students. Unlike prior studies that primarily focus on text messages, static images, or general educational approaches, this review synthesizes evidence on emerging video formats, identifies gaps in current research regarding vaccination behaviors, and offers recommendations for designing more engaging, accessible, and impactful interventions.

## Objective

The aim of this systematic review and meta-analysis was to evaluate the effectiveness of video-based interventions in promoting HPV-related outcomes, such as knowledge, attitudes, beliefs, self-efficacy, vaccination intention, or vaccine initiation and completion among individuals aged 9 to 26.

## Methods

This review followed the Preferred Reporting Items for Systematic Reviews and Meta-Analyses (PRISMA) guidelines to ensure comprehensive and transparent reporting [20].

### Literature search

A master’s-prepared health science librarian and the first author conducted a comprehensive literature search on February 27, 2025, with no date restrictions applied. The bibliographic databases are CINAHL Complete (EBSCOhost), PubMed, Scopus, and PsycINFO, and focused on youth and young adults aged 9–26 years.

The search was modified for each database to incorporate relevant controlled vocabulary, while maintaining a broadly consistent structure across all databases. For example, the complete search strategy in PubMed was ("human papillomavirus" OR "human papillomaviruses" OR HPV OR "Human Papillomavirus Viruses"[Mesh]) AND (Video* OR "Videotape Recording"[Mesh] OR "Video Recording"[Mesh] OR DVD OR videodisc* OR movie OR film OR "digital versatile disc" OR "digital versatile discs" OR audiovisual* OR "audiovisual aids" OR "audiovisual aid" OR "Audiovisual Aids"[Mesh]). Please refer to Appendix 1 for the full search strategy.

### Screening methods and inclusion and exclusion criteria

All identified articles were exported to Covidence for further screening. The screening procedure was carried out in two stages, independently by two reviewers. In the first stage, two authors independently screened the titles and abstracts of the articles. The other two authors then read the full texts to assess eligibility and determine if the studies meet the inclusion criteria. A third reviewer decided on the article’s inclusion in case of disagreement.

To be included, studies must be evaluated as a video-based intervention to promote HPV vaccination among individuals aged 9 to 26. There were no restrictions on the settings or countries of the interventions, and these could be in-person, online, or mobile messaging. A control group was not a necessary requirement for inclusion within this review.

Studies were included if they met the following criteria: (1) a randomized controlled trial (RCT) or a quasi-experimental intervention studies with pre- and post-data with a control group; (2) targeted individuals aged 9 to 26; (3) aimed to improve HPV knowledge, attitudes toward HPV and the vaccine, vaccination self-efficacy, vaccination intention, or vaccine uptake (initiation, completion); and (4) were published in English and peer-reviewed journals. Studies were excluded if they: (1) targeted parents or healthcare providers (even if outcomes were measured in youth or young adults); (2) focused on topics other than HPV and the vaccine (e.g., Pap smears, cervical cancer screening, or sexual abstinence); and (3) involved participants younger than 9 or older than 26 years.

### Data extraction

Data extraction was performed by filling out an Excel sheet with a detailed description of the main information from the selected studies. Two authors also conducted data extraction independently to avoid measurement bias due to misinterpretation of details or the loss of essential data. If information was insufficient, the corresponding authors of the articles were contacted for clarification or additional information.

The following data items were extracted for each study, when available: (a) study identifiers (authors, year of publication), (b) study design characteristics (purpose, sample size, study design, assessment methods (e.g., survey, interview, focus group) type and duration of intervention, and follow-up period, (c) target population items (age, birth sex), (d) setting (online or in-person), and (e) reported outcomes. Authors were contacted if there was any missing data.

### Data synthesis

The primary outcome to be evaluated include the use of video-based interventions to improve: 1) HPV knowledge, 2) attitudes towards HPV and the vaccine, 3) vaccination self-efficacy, 4) vaccination intention, and 5) vaccine uptake. Additional outcomes, such as the overall effectiveness of each intervention, was analyzed. A detailed summary of the results was provided in the text and tables. Key study characteristics include design, sample size, duration, follow-up period, sample demographics (e.g., birth sex), intervention characteristics (e.g., narrative/storytelling, movie), delivery method (e.g., online, in-person), setting e.g., school, community, hospital/clinic, home), drop-out rates, measures, and reported strengths and limitations.

### Meta-analysis

Effect size data were analyzed using Comprehensive Meta-Analysis (Version 4) software to calculate Hedge’s *g* for continuous outcomes (knowledge, attitudes, beliefs, self-efficacy, and intention)*.* Hedges’ *g* was used, as opposed to Cohen’s *d* due to the correction factor for the low number of studies (*k*= 2–6) included in this review to ensure adequate statistical power [21]. With an increase in sample size, Cohen’s *d* and Hedges’ *g* eventually converge. Pooled intervention effect sizes were conducted using random effects models for each outcome using pre- and post- intervention means, standard deviations, sample size for intervention and control groups. For one study, independent t-test statistics, sample size, and p-value were used to calculate Hedge’s *g*. Although quasi-experimental studies with and without control groups were considered, only quasi-experimental studies with control groups met inclusion and exclusion criteria. Therefore, RCT and quasi-experimental studies were included in all random effect models. No unit-of-analysis adjustments were required, as each study contributed a single, independent pre-post effect size per outcome, and no follow-up assessments were reported.

Between-study variance (τ^2^) was estimated using the restricted maximum likelihood (REML) estimator. Statistical heterogeneity of the studies was evaluated using Cochran’s Q and I^2^statistics. A random effects model was used because all interventions most likely would not have the same effect [22]. The effect direction was set to positive indicating that *HPV vaccine* intervention increased participants’ knowledge, attitudes, and beliefs. Studies with a large residual of at least 2.58 and a total change in I^2^greater than 10% were influential outliers [23]. If the total change in I^2^ was greater than 10% and was identified to be an influential outlier, we accepted the removal of said outlier.

### Quality assessment

Two tools were used to evaluate the quality and risk of bias of the included studies: (1) the second version of the Cochrane Risk of Bias tool (RoB 2) for randomized controlled trials and (2) the Risk of Bias In Non-Randomized Studies of—Interventions (ROBINS-I) tool for quasi-experimental studies. The RoB 2 tool evaluates the RCTs using five high-quality domains: (1) risk of bias arising from the randomization process; (2) risk of bias due to deviations from the intended interventions; (3) risk of bias due to missing outcome data; (4) risk of bias in measurement of the outcome; and (5) risk of bias in selection of the reported results (21). Each domain is scored on a scale of low risk of bias, some concerns, and high risk of bias*.*

The ROBINS-I tool assesses quasi-experimental studies using seven high-quality domains: (1) bias due to confounding; (2) bias in selection of participants into the study; (3) bias in classification of interventions; (4) bias due to deviations from intended interventions; (5) bias due to missing data; (6) bias in measurement of outcomes; and (7) bias in selection of the reported results. Each domain is scored on a scale of low risk of bias, moderate risk of bias, serious risk of bias, critical risk of bias, or no information.

### Quality of evidence for each outcome

The Grading of Recommendations, Assessment, Development and Evaluations Tool (GRADE) was used to evaluate the quality of evidence for each HPV outcome (knowledge, attitudes, beliefs, self-efficacy, and intention). The GRADE tool evaluates quality of evidence using the following high-quality domains: study type, overall risk of bias, inconsistency, indirectness, imprecision, and publication bias (See Table 1) [31].

**Table 1:** Quality of evidence assessment for knowledge, attitudes, beliefs, self-efficacy, and intention

**Effect of Videos on HPV knowledge**	**Study type**	**Overall Risk of Bias**	**Inconsistency** There is substantial heterogeneity of the included studies, where I2 was 72.1% and the Q-value was 14.3 (p =.006)	**Indirectness** Poor quality is not due to indirect comparison of the population differences. However, HPV knowledge may be indirectly assessed in the included studies	**Imprecision** Determined as precise because the population is greater than 400 participants	**Publication bias** In funnel plots visual asymmetry was present suggesting publication bias. Using Egger’s test, no publication bias could be identified	**Overall quality of evidence:** For HPV knowledge, very low quality was found
Kim, M. et al., 2020 A storytelling Intervention in a Mobile, Web- Based Platform: A Pilot Randomized Controlled Trial to Evaluate the Preliminary Effectiveness to Promote Human Papillomavirus Vaccination in Korean American College Women [24]	RCT	Some concerns					
A. Krawczyk et al., 2012 How to Inform: Comparing Written and Video Education Interventions to Increase Human Papillomavirus Knowledge and Vaccination Intentions in Young Adults [25]	RCT	High					
Lemos, M. S., et al., 2017 Raising cervical cancer awareness: Analyzing the incremental efficacy of Short Message Service (SMS) combined with video intervention [26]	Quasi	Critical					
Occa, A., et al., 2022 Helping Children to Participate in Human Papillomavirus–Related Discussions: Mixed Methods Study of Multimedia Messages [27]	RCT	High					
Effect of Videos on HPV attitudes	Study type	Overall Risk of Bias	Inconsistency There is substantial to considerable heterogeneity of the three studies that evaluated intervention effects on HPV attitudes (I2 = 83.62%, Q = 18.3, p <.001)	Indirectness Poor quality is not due to indirect comparison of the population differences. However, HPV attitudes may be indirectly assessed in the included studies	Imprecision Determined as imprecise because the population is less than 400	Publication bias In funnel plots visual was symmetric and using Egger’s test, no publication bias could be identified	Overall quality of evidence: For this outcome, very low quality of evidence was found
Kim, M. et al., 2020 A storytelling Intervention in a Mobile, Web- Based Platform: A Pilot Randomized Controlled Trial to Evaluate the Preliminary Effectiveness to Promote Human Papillomavirus Vaccination in Korean American College Women [24]	RCT	Some concerns					
Lismidiati, W. et al., 2020 Human Papillomavirus (HPV) Health Savings as an Alternative Solution: HPV Vaccination Behavior in Adolescents [42]	Quasi	Critical					
Occa, A., et al., 2022 Helping Children to Participate in Human Papillomavirus–Related Discussions: Mixed Methods Study of Multimedia Messages [27]	RCT	High					
Effect of Videos on HPV beliefs	Study type	Overall Risk of Bias	Inconsistency There is substantial to considerable heterogeneity of the two studies that evaluated intervention effects on HPV beliefs (*n* = 2, Q = 4.52, *p* = 0.033, I2 = 77.92%)	Indirectness Poor quality is not due to indirect comparison of the population differences. However, HPV beliefs may be indirectly assessed in the included studies	Imprecision Determined as precise because the population is greater than 400 participants	Publication bias Publication bias analyses were not conducted because only 2 studies evaluated HPV beliefs	Overall quality of evidence: For this outcome, very low quality of evidence was found
Gerend, M. et al., 2013 The Multidimensional Nature of Perceived Barriers: Global Versus Practical Barriers to HPV Vaccination [28]	Quasi	Critical					
Lismidiati, W. et al., 2020 Human Papillomavirus (HPV) Health Savings as an Alternative Solution: HPV Vaccination Behavior in Adolescents [42]	Quasi	Critical					
Effect of Videos on HPV self efficacy	Study type	Overall Risk of Bias	Inconsistency: These studies had a substantial heterogeneity (Q = 67.92, *p* < 0.001, I2 = 92.64%)	Indirectness Poor quality is not due to indirect comparison of the population differences. However, HPV self-efficacy may be indirectly assessed in the included studies	Imprecision Determined as precise because the population is greater than 400 participants	Publication bias In funnel plots visual was asymmetric, indicating that there is some publication bias. However, using Egger’s test was non-significant, indicating no publication bias	Overall quality of evidence: For this outcome, very low quality of evidence was found
Darville-Sanders et al., 2022 Someone You Love” documentary: using narratives in entertainment media to increase HPV vaccination in Georgia [32]	Quasi	Critical					
Hopfer, S. 2011 Effects of a Narrative HPV Vaccination Intervention Aimed at Reaching College Women: A Randomized Controlled Trial [29]	RCT	Some Concerns					
Lemos, M. S., et al., 2017 Raising cervical cancer awareness: Analyzing the incremental efficacy of Short Message Service (SMS) combined with video intervention [26]	Quasi	Critical					
Lismidiati, W. et al., 2020 Human Papillomavirus (HPV) Health Savings as an Alternative Solution: HPV Vaccination Behavior in Adolescents [42]	Quasi	Critical					
Occa, A., et al., 2022 Helping Children to Participate in Human Papillomavirus–Related Discussions: Mixed Methods Study of Multimedia Messages [27]	RCT	High					
Effect of Videos on HPV Intention	Study type	Overall Risk of Bias	Inconsistency With a Q-statistic of 94.95 (p <.001) and an I2 of 91.57%, there was substantial to considerable heterogeneity between the studies	Indirectness Poor quality is not due to indirect comparison of the population differences. However, HPV intention may be indirectly assessed in the included studies	Imprecision Determined as precise because the population is greater than 400 participants	Publication bias In funnel plots visual was asymmetric, indicating that there is some publication bias. However, using Egger’s test was non-significant, indicating no publication bias	Overall quality of evidence: For this outcome, very low quality of evidence was found
Darville-Sanders et al., 2022 Someone You Love” documentary: using narratives in entertainment media to increase HPV vaccination in Georgia [32]	Quasi	Critical					
Hopfer, S. 2011 Effects of a Narrative HPV Vaccination Intervention Aimed at Reaching College Women: A Randomized Controlled Trial [29]	RCT	Some Concerns					
A. Krawczyk et al., 2012 How to Inform: Comparing Written and Video Education Interventions to Increase Human Papillomavirus Knowledge and Vaccination Intentions in Young Adults [25]	RCT	High					
Leader, A. E., et al., 2022 The impact of HPV vaccine narratives on social media: Testing narrative engagement theory with a diverse sample of young adults [30]	Quasi	Critical					
Lemos, M. S., et al., 2017 Raising cervical cancer awareness: Analyzing the incremental efficacy of Short Message Service (SMS) combined with video intervention [26]	Quasi	Critical					
Occa, A., et al., 2022 Helping Children to Participate in Human Papillomavirus–Related Discussions: Mixed Methods Study of Multimedia Messages [27]	RCT	High					

For RCTs, we rate the baseline quality “high”; and for quasi-experimental studies, we rated the baseline quality “low”. For each subsequent domain, if no serious concern exists, we did not downgrade the evidence quality. If serious concerns exist, we downgraded the evidence by one level. If very serious concern exists, we downgraded the evidence by two levels. Overall risk of bias refers to the risk of bias assessments conducted using the RoB2 and ROBINS-I tools. Inconsistency refers to any heterogeneity, informed using I^2^ and the Q-statistics, that is present between the included articles. Indirectness refers to study components (population, intervention, comparator, or outcomes) that indirectly answer the review question. Imprecision refers to the lack of participants in the included studies that estimate the total effect size. For continuous variables, a total sample of less than 400 is deemed insufficient. Lastly, publication bias refers to the reported publication bias statistics from calculating the pooled effect sizes. The two statistics used to inform publication bias include the funnel plots and Egger’s test.

## Results

### Study selection

**Fig. 1:**
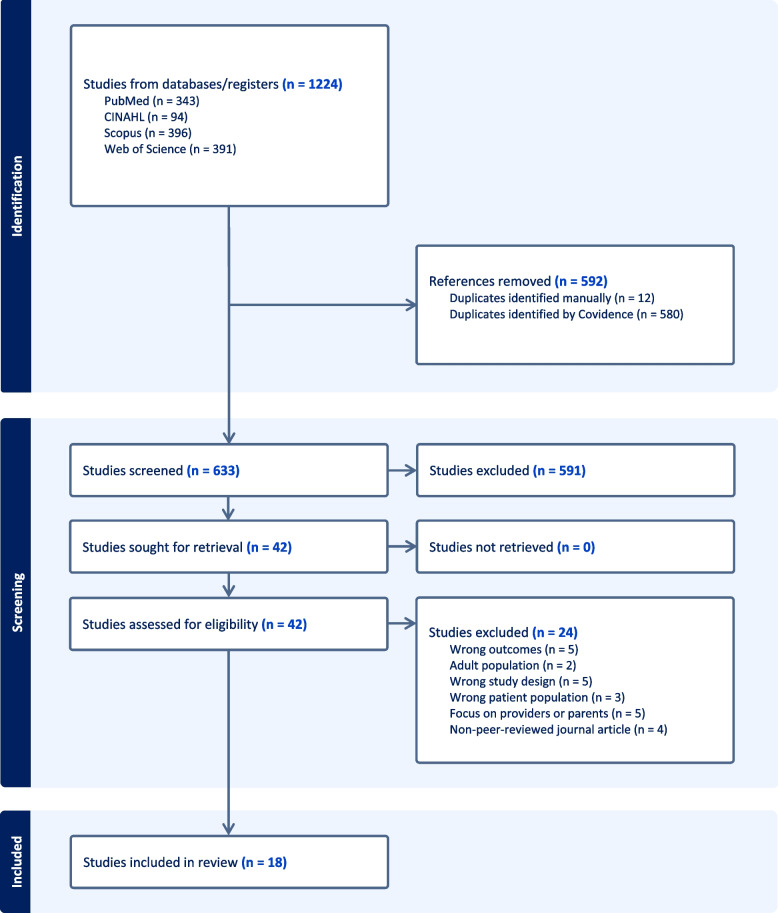
PRISMA flow diagram of study selection for HPV video interventions

Our search strategy initially identified 1,224 publications (Fig. 1). After screening, 42 articles were assessed for full-text eligibility. Of these, 24 articles were excluded for the following reasons: wrong outcomes (*n* = 5), adult populations (*n* = 2), wrong study designs (*n* = 5), wrong patient population (*n* = 3), focus on providers or parents (*n* = 5), and non–peer-reviewed journal articles (*n* = 4). Ultimately, 18 articles met the inclusion criteria and were included in the final review.

### Study characteristics

Of the 18 articles in the final analysis (See Supplementary Table 1. The Summary Table), most were conducted in the United States (k = 11, 61.1%) [24, 28–30, 32–38]. The remaining studies were each performed in a different country: Canada (k = 1, 5.6%), the United Kingdom (k = 1, 5.6%), Saudi Arabia (k = 1, 5.6%), Turkey (k = 1, 5.6%), Portugal (k = 1, 5.6%), Indonesia (k = 1, 5.6%), and Italy (k = 1, 5.6%) [25–27, 39–42]. Regarding study design, 7 were randomized controlled trials (RCTs) and 11 were quasi-experimental studies.

### Sample characteristics

Eight studies focused on female participants in college [24, 26, 28–30, 34, 40] and junior high school settings [42]. Two studies targeted parent–daughter dyads [37, 39] and one study focused specifically on male college students [36]. Four studies included both male and female participants from middle school [27], high school [38] or college populations [25, 32]. Additionally, two studies examined women attending OB/GYN outpatient clinics [35, 41] and one study focused on women in community-based settings [32].

A total of 5,873 participants (an average of 326.3 participants per study) were included in this review. Eleven of the studies reported age range, and six of the studies reported mean age. Of the studies that reported age range, participants were 11 to 45 years old. On average, participants were aged 13.3 to 22 years old. One study recruited “pre-adolescent participants” but did not include age range or mean age. The majority of the participants were female (*n* = 4956; 84.4%). Male participants comprised 2.5% of the study sample (*n* = 165). The racial and ethnic demographics of the included participants were white (*n* = 2107, 35.9%), black (*n* = 777, 13.2%), Saudi Arabic Middle Eastern (*n* = 535, 9.1%), Hispanic (*n* = 489, 8.3%), Asian (*n* = 425, 7.2%), Other races (*n* = 203, 3.5%), Mixed races (*n* = 78, 1.3%), Native Hawaiian or Pacific Islander (*n* = 9, 0.2%), American Indian (*n* = 7, 0.1%), Not reported (*n* = 1342, 22.9%).

### Intervention characteristics

Two studies conducted follow-up assessments two months after the interventions to evaluate HPV vaccine initiation [29, 32], while another two studies followed participants for 9–10 months to assess vaccine series completion [24, 33]. Other studies assessed outcomes using only pre- and post-intervention measurements.

The included interventions’ duration ranged from a 5-min video to 6 months long. Five interventions did not report the length of their intervention (*k* = *5*) [25, 27, 28, 34, 39]. Of the eighteen studies, twelve of which included a comparison group: class or treatment as usual (*k* = *5*) [32, 35, 38, 39, 42], another video type (*k* = 2; [narrative vs. non narrative: *k* = 1 [29] and positive messaging vs. neutral messaging vs negative messaging: *k* = *1*] [28], pamphlet or written intervention: *k* = 5 [24, 25, 35, 37, 41]; health care professional guest speaker: *k* = 1 [41]; text messaging: *k* = 1 [26] Eleven studies reported a theoretical framework in which their interventions were based upon: Health Belief Model (*k*= 3) [25, 32, 36]. Transportation-Imagery Model (k = 1) [32], Exemplification theory *k* = 1) [29], Situation-specific theoretical framework (*k* = 1) [24]; Information, Motivation, Behavioral Skills Model (*k* = 4); Narrative Engagement Theory (*k* = 1) [30]; Revised Network Model (*k* = 1) [37]; The Storytelling Narrative Communication Theory (*k* = 1) [37]; Theory of Planned Behavior (*k* = 1) [27]; Social Cognitive Theory (*k* = 1) [27]; Authors developed own model *(k*= 2) [28, 39].

### Intervention type

We classified the 18 studies into three categories: informational videos [25, 28, 32, 34, 38, 39, 41, 42], narrative/storytelling interventions [24, 29, 30, 32, 37], and videos combined with other strategies [26, 27, 36, 40]. Informational HPV videos are generally fact-based and presented in a format similar to lectures or public service announcements. They deliver straightforward educational content, focusing on statistics and explanations of HPV transmission, prevention strategies, and vaccine efficacy. In contrast, storytelling interventions convey key messages through personal narratives. These stories, shared by relatable characters or drawn from real-life experiences, are often emotionally engaging and culturally relevant, helping the audience connect more deeply with the message.

Among the nine interventions utilized informational videos. The content of these videos focused on providing factual information regarding HPV, such as disclosing the risks associated with HPV, including cervical cancer and other HPV-related health consequences. These videos also encouraged participants to consider the efficacy and safety of HPV vaccination and/or Pap tests. Five studies in the narrative/storytelling category, video durations ranged from 1.5 min to 1.5 h and featured diverse women diagnosed with HPV infection or cervical cancer [2, 24, 29, 30, 37]. Particularly, two studies featured culturally tailored groups, with one study featuring Korean American multigenerational women [24] and another highlighting Khmer [37] mothers and daughters. The remaining studies used story-based videos featuring women with HPV infection, medical providers, or peers to highlight risk factors associated with HPV and to promote awareness, knowledge, and positive attitudes toward HPV infection and the HPV vaccine. The remaining four studies combined video-based interventions with additional strategies—such as social media campaigns with videos, Facebook sharing, posters, mobile text messages paired with video content, and animated videos integrated into a web-based game—to promote HPV vaccine knowledge, intention, and uptake [26, 27, 36, 40].

### Intervention effects

#### Meta-analysis for continuous outcomes

A total of 9 articles reported data used to calculate total effect sizes [24–27, 29, 30, 32, 34]. The remainder of the studies reported prevalence. Meta analysis was conducted for HPV knowledge, attitudes, beliefs, self-efficacy, and vaccination intention outcomes.

##### HPV Knowledge

**Fig. 2:**
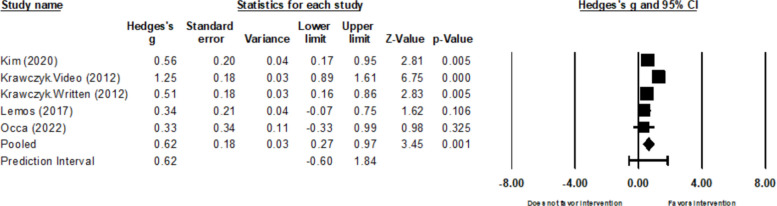
Forest plot for HPV knowledge

Ten out of 18 reports described data about HPV knowledge (participants’ knowledge of HPV infection and/or the HPV vaccine as outcomes) [24–27, 29, 35, 38–40, 42]. Four (one study reported two interventions; *k*= 5) out of 10 studies provided meta-analytic data used to calculate intervention effects of HPV knowledge [24–27]. The between-study variance (τ^2^) was 0.11. There is substantial heterogeneity of the included studies, where I^2^ was 72.1%. The *Q*-value was 14.3 (*p* = 0.006). No influential outliers were identified. The combined pooled effect size for this outcome was *g* = 0.62 (95% CI: 0.27 to 0.97; Fig. 2). Of the remaining six studies, five that utilized video interventions showed increased actual or perceived HPV knowledge [35, 38–40, 42].

##### Attitudes

**Fig. 3:**
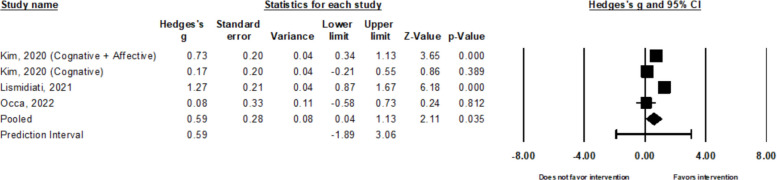
Forest plot for HPV attitudes

There is substantial to considerable heterogeneity of the three studies (four effect sizes) that evaluated intervention effects on HPV attitudes (*k* = 4, τ^2^ = 0.25, I^2^ = 83.62%, *Q* = 18.3, *p* < *0.0*01) [24, 27, 42]. There were no influential outliers. The pooled intervention effects on HPV attitudes were moderate (*g* = 0.59, 95% CI: 0.04 to 1.13; Fig. 3).

##### Beliefs

**Fig. 4:**
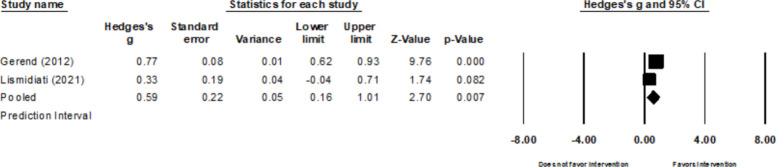
Forest plot for HPV beliefs

Like HPV attitudes, there is substantial to considerable heterogeneity of the two studies that evaluated intervention effects on HPV beliefs (*k* = 2, τ^2^ = 0.08, *Q* = 4.53*, p* = 0.033*,* I^2^= 77.92%) [26, 34]. No influential outliers were identified. The pooled intervention effects for HPV beliefs were 0.59 (95% CI: 0.05, 1.01; Fig. 4). One study indicated that there were improvements in health beliefs related to susceptibility, perceived severity, self-efficacy, subjective norms, and barriers [36].

##### Self-efficacy

**Fig. 5:**
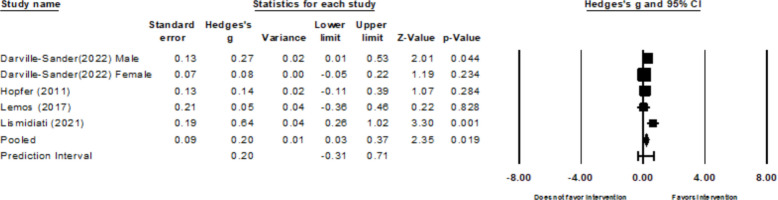
Forest plot for HPV self-efficacy

Six out of 18 studies reported on HPV related self-efficacy [26, 27, 29, 32, 36, 42]. *Five of these studies*(six effect sizes because one study described intervention effects for males and females separately) described vaccine self-efficacy data used to calculate the pooled effect size [26, 27, 29, 32, 42]. These studies had a substantial heterogeneity (*k* = *6,* τ^2^ = 0.26*, Q* = 67.92*, p* < 0.001*,* I^2^= 92.64%). There was one influential outlier with a residual of 6.67 [26].. The overall pooled effect size for HPV self-efficacy is *g* = *0.*23 (*95%CI:* 0.10 to 1.02; Fig. 5). After the removal of this influential outlier, the I^2^ reduced to 51.8% (*k* = *5,* τ^2^ = 0.02, *Q* = 8.30, *p* = 0.081). The new pooled effect size is 0.20 (95% CI: −0.31to 0.71). The only study that did not include data to calculate effect size reported a significant increase in self-efficacy after receiving the video-intervention [36].

##### Intention

**Fig. 6:**
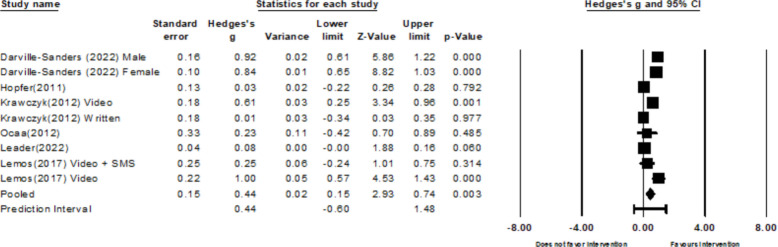
Forest plot for HP intention

Nine out of 18 studies reported vaccination intentions as the outcome [25–30, 32, 33, 37]. Six of the studies—two of which described two separate interventions, and one that reported intervention effects separately for males and females—provided HPV vaccination intention data used in the meta-analysis [3, 25–27, 29, 32]. The variance between all of the included studies was 0.18 *(k* = 9)*.* With a Q-statistic of 94.95 *(p* < *0.001*) and an I^2^ of 91.57%, there was substantial to considerable heterogeneity between the studies. The pooled effects for HPV vaccine intention, are small to moderate (*g* = 0.44; 95% CI: 0.15 to 0.74; Fig. 6). Of the remaining 3 studies, 37.73% of participants (412 intended/192 total) disclosed that they plan to receive the HPV vaccine [33–35].

### Intervention effects for categorical outcomes

#### Uptake (Initiation and Completion) of HPV vaccine

Seven studies reported on the prevalence of HPV uptake/initiation [24, 28, 29, 32, 34, 36, 37] and two studies [33, 34] reported on the completion of the HPV vaccination series. HPV vaccine uptake among participants showed that a small proportion had initiated or completed the series: 8.84% (193 out of 2,184) had started the vaccine, while 13.67% (148 out of 1,083) had completed the full series.

### Instruments used to assess HPV outcomes

Researchers employed a variety of instruments for data collection. Each study either reported a Cronbach’s alpha value or indicated that the instruments had been previously used and validated in earlier research. Most instruments demonstrated strong internal consistency, with Cronbach’s alpha values of 0.80 or higher, and only two studies reporting values below 0.80 [28, 32]. The majority of instruments measured participants’ knowledge of, and attitudes toward, the HPV vaccine and cervical cancer. One study utilized two instruments developed based on constructs from the Theory of Planned Behavior and components of the Health Belief Model [33]. Because the included studies primarily aimed to evaluate the effectiveness of their developed interventions in promoting HPV vaccination uptake or improving HPV-related knowledge, attitudes, and/or intentions, none of them reported construct validity.

### Intervention effects based on intervention type

Among the nine studies in the informational video category, one study assessed HPV vaccine completion and reported that 43% of participants in the intervention group and 32% in the control group completed the three-dose series [33]. Six studies demonstrated significant improvements in knowledge, vaccine acceptance, and/or intention to receive the HPV vaccine [25, 27, 35, 39, 41, 42]. However, two studies found no significant differences between the intervention and control groups [28, 34].

Of the five narrative/storytelling interventions, four studies measured vaccine initiation [24, 29, 30, 32, 37]. Three reported significantly higher initiation rates in the intervention groups than controls. In contrast, one study found no difference in vaccine uptake between groups [24, 29, 32, 37]. The remaining study found that greater video engagement was associated with stronger intentions to discuss receiving the HPV vaccine [37].

All four studies in the videos combined with other strategies category reported significant improvements in participants’ knowledge about HPV and the HPV vaccine and in perceived susceptibility, perceived severity, and self-efficacy related to vaccination [26, 27, 36, 40]. However, one study found that while participants’ knowledge about cervical cancer significantly improved, their intention to receive the HPV vaccine did not increase [26].

#### Publication bias

**Fig. 7:**
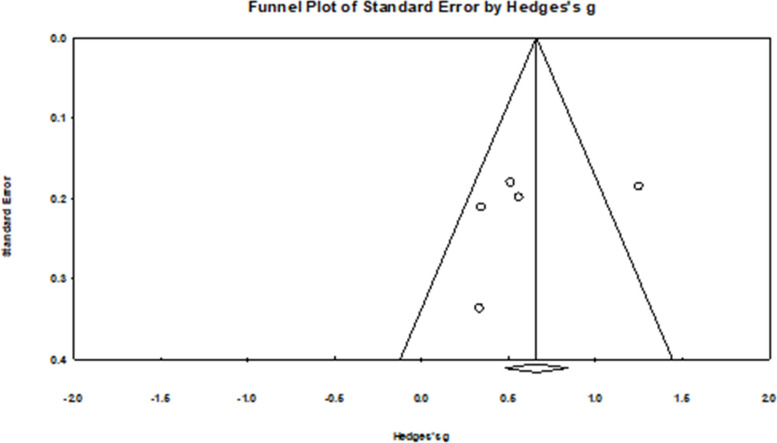
Funnel plot for knowledge

**Fig. 8:**
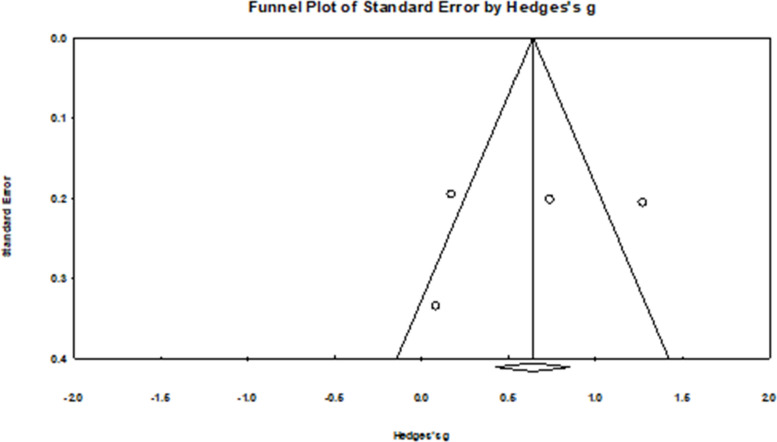
Funnel plot for attitudes

**Fig. 9:**
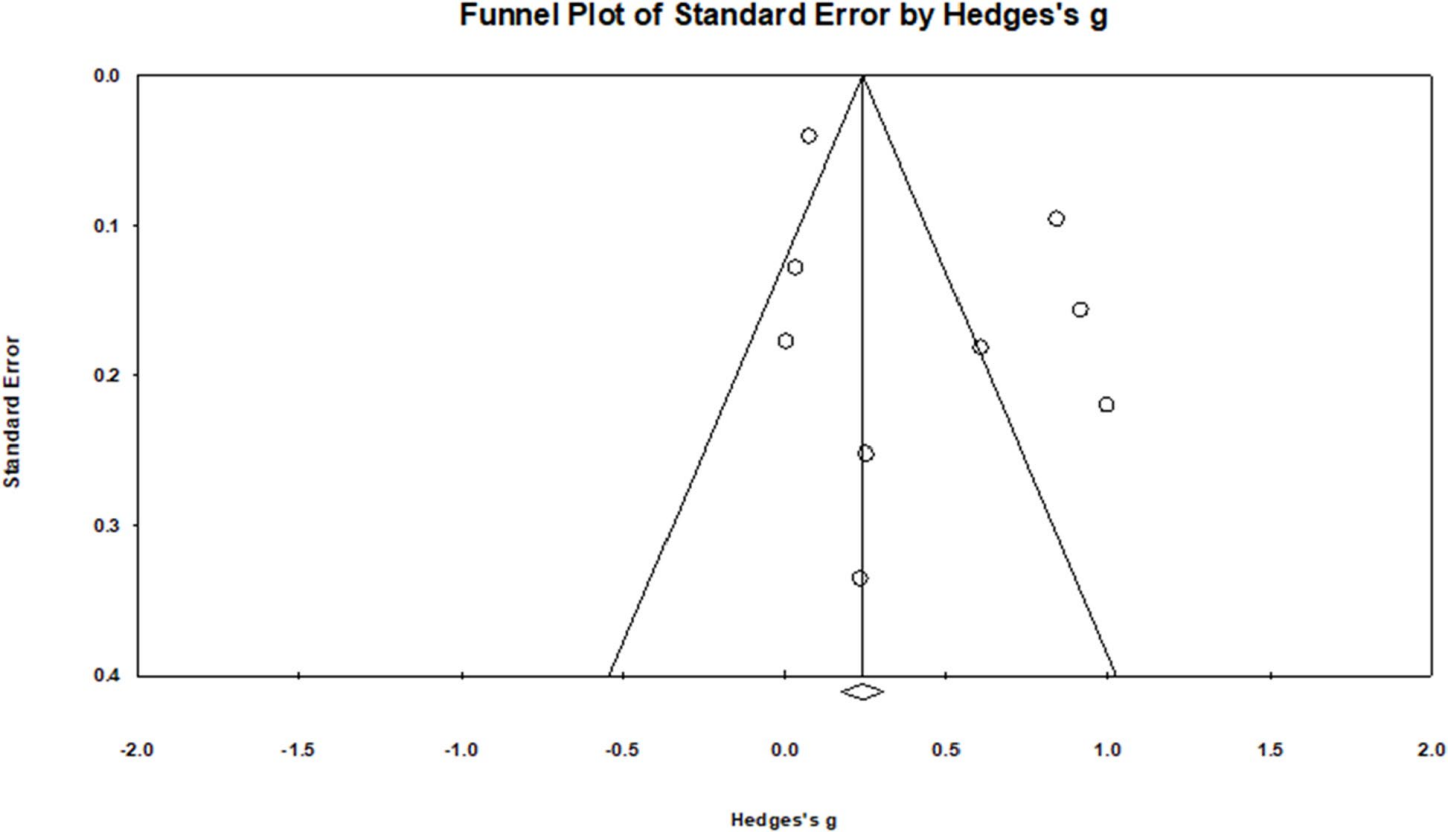
Funnel plot for HPV Intention

**Fig. 10:**
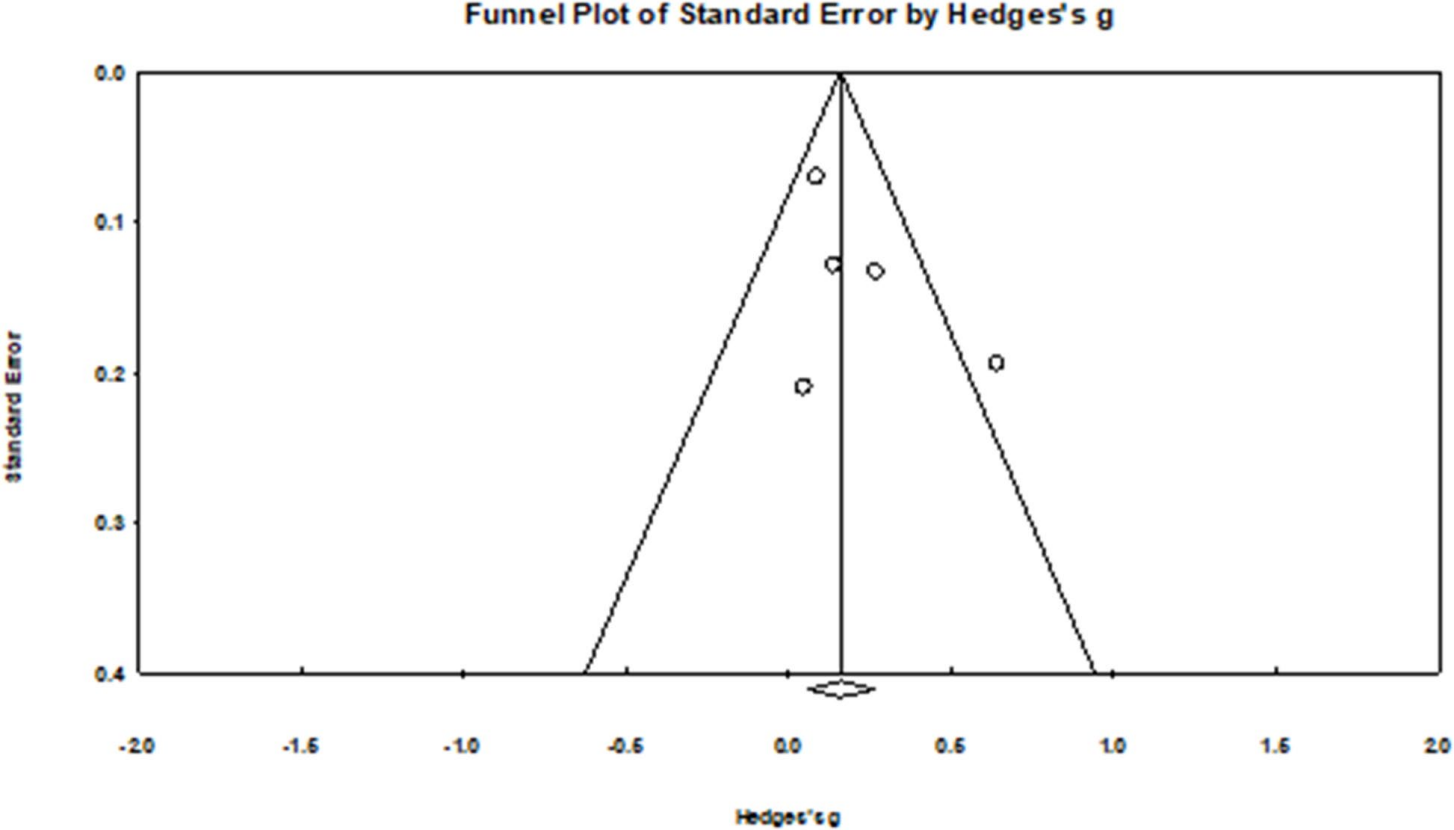
Funnel plot for self-efficacy

For the HPV knowledge outcome, results from Begg and Mazumdar’s test (Tau = −0.40, *p* = 0.327) and Egger’s test (b = −3.45; 95% CI: −18.35 to 11.46, *p* = 0.515) indicated no firm evidence of publication bias. However, the funnel plot was asymmetric upon visual inspection (see Fig. 7). However, due to the low sample size, no correction was performed. There was no strong evidence of publication bias for HPV attitudes (*Tau* = 0.33, *p* = 0.497; *b* = −3.65, 95%CI: −36.13 to 28.72, *p* = 0.676, see Fig. 8), HPV intentions (Tau = 0.03, *p* = 0.917; *b* = *2.66*, 95%CI: −1.42 to 6.74, *p* = 0.167), and HPV self-efficacy (*Tau* = 0.40, *p* = 0.327; *b* = 1.97, 95% CI: −2.77 to 6.71, *p* = 0.278). However, after visual inspection of the funnel plots for HPV intentions (Fig. 9) and self-efficacy (Fig. 10), they did not appear symmetric. After performing Duval and Tweedie trim and fill, Duval and Tweedie’s trim and fill did not advise trimming studies for random effects models. Therefore, no studies were removed in the calculation of the final pooled effect size for these outcomes.

### Quality assessment of studies and outcomes

Seven RCTs were evaluated using the second version of the Cochrane Risk of Bias tool (RoB 2; See Table 2). Eleven quasi-experimental studies were evaluated using the Risk Of Bias In Non-Randomized Studies of—Interventions (ROBINS-I) tool. Of the seven studies assessed using the RoB 2 tool, three (42.9%) were rated to have some risk and three (42.9%) had a high risk of bias. Some and high risks of bias arose due to bias from the randomization process (*n* = 6, 85.7%), deviations from the intended interventions (*n* = 5, 71.4%), missing outcome data (*n* = 2, 28.6%), and measurement of the outcomes (*n* = 3, 42.9%).

**Table 2:** Risk of bias table of the included studies

Randomized control trials											
	Risk of bias arising from the randomization process		Risk of bias due to deviations from the intended interventions		Risk of bias due to missing outcome data			Risk of bias in measurement of the outcome		Risk of bias in selection of the reported result	
Cory, 2019 [35]	Some concerns		Some concerns		Low of bias			High of bias		Low of bias	
Lee 2018 [37]	Some concerns		Some concerns		Some concerns			High of bias		Low of bias	
Gerend, 2012 [34]	Some concerns		Some concerns		Low of bias			Low of bias		Low of bias	
Hopfer, 2011 [29]	Some concerns		Low of bias		Low of bias			Low of bias		Low of bias	
Kim, 2020 [24]	Some concerns		Some concerns		Some concerns			Low of bias		Low of bias	
Krawczyk, 2012 [25]	Some concerns		Some concerns		Low of bias			High of bias		Low of bias	
Vanderpool, 2013 [33]	Low of bias		Low of bias		Low of bias			Low of bias		Low of bias	

Quasi-experimental studies											
	Bias due to confounding	Bias in selection of participants into the study		Bias in classification of interventions		Bias due to deviations from intended interventions	Bias due to missing data		Bias in measurement of outcomes		Bias in selection of the reported result
Al-Shaikh et al., 2017 [40]	Serious risk of bias	Critical risk of bias		Serious risk of bias		Low risk of bias	Critical risk of bias		Moderate risk of bias		Moderate risk of bias
Brabin, 2010 [39]	Serious risk of bias	Critical risk of bias		Serious risk of bias		Low risk of bias	Critical risk of bias		Serious risk of bias		Serious risk of bias
Darville-Sanders, 2022 [32]	Low risk of bias	Critical risk of bias		Moderate risk of bias		Low risk of bias	Critical risk of bias		Moderate risk of bias		Moderate risk of bias
Ekmez & Ekmez, 2022 [41]	Low risk of bias	Low risk of bias		Moderate risk of bias		Low risk of bias	Low risk of bias		Serious risk of bias		Serious risk of bias
Gerend, 2013 [28]	Low risk of bias	Low risk of bias		Moderate risk of bias		Low risk of bias	Critical risk of bias		Serious risk of bias		Serious risk of bias
Hughes, 2020 [36]	Serious risk of bias	Low risk of bias		Moderate risk of bias		Low risk of bias	Critical risk of bias		Serious risk of bias		Serious risk of bias
Leader, 2022 [30]	Serious risk of bias	Critical risk of bias		Moderate risk of bias		Low risk of bias	Critical risk of bias		Moderate risk of bias		Moderate risk of bias
Lemos, 2017 [26]	Serious risk of bias	Critical risk of bias		Moderate risk of bias		Moderate risk of bias	Low risk of bias		Moderate risk of bias		Moderate risk of bias
Lismidiati, 2021 [42]	Low risk of bias	Low risk of bias		Moderate risk of bias		Critical risk of bias	Low risk of bias		Serious risk of bias		Serious risk of bias
Merzouk, 2011 [38]	Serious risk of bias	Low risk of bias		Moderate risk of bias		Moderate risk of bias	Critical risk of bias		Moderate risk of bias		Moderate risk of bias
Occa, 2022 [27]	Serious risk of bias	Low risk of bias		Moderate risk of bias		Moderate risk of bias	Critical risk of bias		Moderate risk of bias		Moderate risk of bias

For the remaining eleven quasi-experimental studies, 2 (18.2%) had a serious risk of bias, and the remaining 9 (81.8%) were rated to have a critical risk of bias. Critical risks of bias were due to bias from the selection of participants into the study (*n* = 5, 45.5%), due to deviations from intended interventions, specifically starting and adhering to the intervention (*n* = 1, 9.1%), and missing data (*n* = 8, 72.7%). The remaining 2 quasi-experimental studies had serious concerns for bias in the measurement of outcomes and selection of the reported results.

In addition to conducting quality appraisals of the studies included, quality appraisals of the reported outcomes were also performed. All outcomes (knowledge, attitudes, beliefs, self-efficacy, and intentions) were very low in quality. Reasons for downgrade of outcome quality include (1) the inclusion of quasi-experimental studies, (2) moderate to critical risk of bias for the included studies, and (3) asymmetric funnel plots for HPV knowledge, self-efficacy, and intention.

## Discussion

The purpose of this study was to systematically review the literature on the use of video-based interventions to promote HPV-related outcomes, such as HPV vaccination knowledge, attitude, belief, self-efficacy, vaccination intention, and initiation. Although studies have examined the effectiveness of various interventions to improve HPV-related outcomes, video-based approaches, due to their engaging and accessible nature, may be particularly well-suited for younger populations, such as college students [43–48].

While the use of video-based interventions in HPV-related health promotion is still relatively new, emerging evidence suggests significant promise [43, 47]. Most existing research has focused on traditional formats such as text messages and static images, with limited attention to narrative or storytelling videos commonly found on social media [43–49]. Although current studies support the effectiveness of video- based interventions in increasing HPV knowledge and vaccination intentions among college students, differences in study design, population focus, and outcome measures remain. Randomized trials provide causal evidence but often rely on small, single-site samples with short follow-up periods and primarily assess intentions rather than actual vaccine initiation and completion. Systematic reviews and meta-analyses offer broader trends but face heterogeneity in interventions and populations, limiting generalizability. Future research should examine diverse video formats, include larger, multi-site trials with longer follow-up, and assess actual vaccine uptake to strengthen evidence and identify the most effective strategies for engaging younger populations.

The 18 studies were categorized into informational videos, narrative/storytelling videos, and videos combined with other strategies. Overall, interventions across all three categories demonstrated effectiveness in promoting HPV-related outcomes. Notably, studies utilizing narrative/storytelling videos consistently produced stronger results than those using other formats. Most studies reported significant improvements in HPV vaccination uptake, intentions, acceptability, knowledge, attitudes, and self-efficacy. Narrative or storytelling videos may outperform purely informational approaches because they foster emotional engagement and identification with characters, mechanisms described by the Transportation-Imagery Model and Narrative Communication Theory [50, 51]. These frameworks suggest that when viewers become transported into a story, they experience reduced counterarguing and stronger personal relevance, leading to greater attitude and behavior change. Such immersive engagement may be particularly effective in shaping vaccination intentions among younger audiences, who are accustomed to narrative-driven content on digital and social media platforms [17, 33, 36, 52]. Further research is needed to explore the factors contributing to the effectiveness of narrative/storytelling formats, their influence on HPV vaccine series completion, and how video-based interventions can be optimized and tailored for diverse populations and settings. However, only two studies evaluated completion of the full three-dose HPV vaccine series among females aged 18–26, highlighting the need for further investigation into the factors influencing series completion—an essential element for achieving full vaccine efficacy [10, 11]. Moreover, two studies either did not demonstrate significant improvements in vaccine uptake compared to control groups or failed to meaningfully enhance participants’ knowledge. These mixed findings indicate that, although video-based interventions show overall promise, their effectiveness may vary based on the specific content and implementation strategies employed. This emphasizes the importance of systematically evaluating intervention components and conducting further research to identify and refine the most effective approaches for improving HPV-related outcomes.

The gender imbalance observed across included studies—most of which focused on female participants—raises important considerations for intervention design. Given that HPV affects all genders, future research should intentionally target male populations to address persistent disparities in awareness, perceived susceptibility, and vaccination uptake [25, 28, 31, 33]. Tailored narratives featuring relatable male characters and contexts could help normalize HPV vaccination among men and promote gender-inclusive public health messaging.

This review highlights the importance of incorporating culturally relevant and emotionally engaging video content into public health education and university-based vaccination campaigns. Embedding such interventions within social media platforms can enhance accessibility and engagement among younger populations [43]. Scaling video-based interventions through platforms like YouTube, TikTok, and Instagram offers a cost-effective and sustainable avenue for broad dissemination, particularly when content is optimized for short-form, narrative-driven, and culturally tailored messaging [53–55].

At the policy level, these findings support the integration of video-based strategies into national HPV vaccination initiatives. Collaborative efforts among public health agencies, educational institutions, and digital media platforms can promote consistent, evidence-based messaging and ensure equitable access across diverse populations. Institutional and policy support for digital innovation in vaccine promotion may ultimately strengthen public health outreach, improve vaccination uptake, and reduce HPV-related health disparities.

### Pooled effect size

Our study's pooled effect sizes for outcomes such as HPV knowledge, attitudes, beliefs, self-efficacy, and intentions ranged from minor to moderate *(g* = 0.16 to 0.62)*.*Compared to other meta-analyses evaluating interventions to increase HPV knowledge and awareness, video-based interventions demonstrated relatively stronger effects. For example, a meta-analysis of 34 studies assessing educational and reminder-based digital interventions reported pooled effect sizes ranging from 0.1 to 0.5 [56]. Another meta-analysis of 32 studies examining provider-based interventions found pooled effect sizes ranging from 0.1 to 0.2 [57]. These findings support the potential of video-based interventions as a more impactful strategy for increasing HPV-related knowledge and promoting vaccination. However, given that only nine studies were included in our meta-analysis, further investment is warranted to develop and evaluate video-based HPV interventions.

### Limitations

Video-based interventions represent a relatively new strategy for promoting HPV vaccination uptake. In this review, only 18 studies met the inclusion criteria. Additionally, for the calculation of effect sizes for knowledge, attitudes, beliefs, self-efficacy, and vaccination intention; two to six studies were used. This is primarily due to the fact that many studies did not evaluate HPV vaccination outcomes using valid and reliable measures. Furthermore, multiple follow-up periods were considered, however, they were only reported for vaccination uptake outcomes. Due to the limited number of eligible studies and considerable heterogeneity among them, we were unable to conduct moderation analyses. Additionally, with such a small number of studies that reported each outcome, publication-bias tests were not sufficiently powered. The study variation described in heterogeneity, reliability of publication tests, and funnel plot asymmetric could be due to chance [58].

Another limitation is that effect sizes were derived using available summary statistics (means, SDs, and sample sizes). The reported effect sizes are unadjusted group-level statistics rather than fully adjusted models. As a result, some intervention effects may reflect baseline group differences rather than true causal effects. The considerable heterogeneity and high overall risk of bias calls for more cautious interpretations of the results. This underscores the need for more rigorous and standardized research to facilitate a comprehensive evaluation of intervention effectiveness across diverse populations, settings and delivery approaches.

The low observed publication bias supports the appropriateness of conducting the meta‑analysis and strengthens the credibility of the findings. Notably, most studies were conducted in high‑income countries, particularly the United States, highlighting limited geographic diversity. Indeed, global data indicate that many lower‑ and middle‑income countries report very low HPV vaccination coverage—for example, full‑dose coverage in girls aged 9–14 years was estimated at only ~ 20% globally, and in some countries first‑dose coverage was below 5% [59]. Combined with small sample sizes and high heterogeneity in study design and interventions, this restricts the generalizability of the results to broader populations. Consequently, future research should examine video‑based HPV vaccination interventions in diverse geographic and cultural contexts—including regions with low HPV vaccination rates and limited research—to determine their effectiveness across different health systems, socioeconomic settings, and cultural groups.

## Conclusion

This systematic review and meta-analysis examined the effectiveness of video-based interventions in promoting HPV-related outcomes, including knowledge, attitudes, beliefs, self-efficacy, intentions, and uptake. The findings indicate that video-based interventions, whether used alone or combined with other strategies such as social media campaigns, gamified content, or interactive elements, can significantly improve participants’ knowledge, positively shape attitudes and intentions, and contribute to increases in vaccine uptake. The overall effect sizes for knowledge, attitudes, beliefs, and intentions were small to moderate, supporting the value of video-based strategies as engaging and accessible tools, particularly for younger populations. However, the small effect size observed for self-efficacy and the limited number of studies examining vaccine series completion highlight critical gaps. Additionally, mixed results from multi-component interventions suggest that quality of the content and implementation context are critical factors to their success.

Future research should focus on optimizing randomized controlled trial designs, incorporating long-term follow-up to assess vaccine series completion, and developing more targeted approaches to enhance self-efficacy. Additionally, studies should examine how specific video components—such as narrative structure, message framing, and cultural tailoring—influence HPV vaccine initiation and completion across diverse populations and settings. Applying behavioral theory to the design of video-based interventions, supported by broader policy and institutional efforts, can enhance both their effectiveness and sustainability. Collectively, these efforts are essential to fully realize the potential of video-based strategies in promoting HPV vaccination and improving public health outcomes.

## Supplementary Information

Supplementary Material 1.

Supplementary Material 2. Table 1. Summary Table. Article 1: “Someone You Love” documentary: using narratives in entertainment media to increase HPV vaccination in Georgia [23]. Article 2: Effects of a narrative HPV vaccination intervention aimed at reaching college women: a randomized controlled trial [31]. Article 3: A storytelling intervention in a mobile, Web-based platform: a pilot randomized controlled trial to evaluate the preliminary effectiveness to promote human Papillomavirus vaccination in Korean American college women [32]. Article 4: 1-2-3 Pap” intervention improves HPV vaccine series completion among Appalachian women [29]. Article 5:How to inform: comparing written and video education interventions to increase human papillomavirus knowledge and vaccination intentions in young adults [18]. Article 6: Predicting Human Papillomavirus Vaccine Uptake in Young Adult Women: Comparing the Health Belief Model and Theory of Planned Behavior [24]. Article 7: Effectiveness of health education programme: Level of knowledge about prevention of cervical cancer among Saudi female healthcare students [38]. Article 8: Survey of girls’ recall of a film providing information on human papillomavirus and cervical cancer 6 months after an offer of vaccination [37]. Article 9: Effects of Educational Interventions on Human Papillomavirus Vaccine Acceptability A Randomized Controlled Trial [33]. Article 10: Effects of information sources in HPV vaccine acceptance: prospective randomized trial [25]. Article 11: The Multidimensional Nature of Perceived Barriers: Global Versus Practical Barriers to HPV Vaccination [34]. Article 12: Designing and Implementing an Educational Social Media Campaign to Increase HPV Vaccine Awareness among Men on a Large College Campus [35]. Article 13: The impact of HPV vaccine narratives on social media: Testing narrative engagement theory with a diverse sample of young adults [28]. Article 14: Using narrative intervention for HPV vaccine behavior change among Khmer mothers and daughters: A pilot RCT to examine feasibility, acceptability, and preliminary effectiveness [36]. Article 15: Raising cervical cancer awareness: Analysing the incremental efficacy of Short Message Service (SMS) combined with video intervention [39]. Article 16: Human Papillomavirus (HPV) Health Savings as an Alternative Solution: HPV Vaccination Behavior in Adolescents [40]. Article 17: Knowledge of HPV in West Virginia High School Health Students and the Effects of an Educational Tool [30]. Article 18: Helping Children to Participate in Human Papillomavirus–Related Discussions: Mixed Methods Study of Multimedia Messages [41].

## Data Availability

The data supporting the findings of this study are available from the corresponding author upon reasonable request. Data access is subject to the authors’ approval.

## Abbreviation

HPV: Human Papillomavirus vaccine
CDC: The Centers for Disease for Disease Control and Prevention
RCT: Randomized Controlled Trial
PRISMA: The Preferred Reporting Items for Systematic Reviews and Meta-Analyses
RoB 2: The Cochrane Risk of Bias tool for randomized controlled trials
ROBINS-I: The Risk of Bias In Non-Randomized Studies of—Interventions
GRADE: The Grading of Recommendations, Assessment, Development and Evaluations
