# Supplementary material for: Video-based interventions to promote HPV vaccination among individuals aged 9 to 26: a systematic review and meta-analysis
Source: BMC Public Health. 2026 Mar 14;26:1308. doi: 10.1186/s12889-026-26759-w (PMC13101323; doi:10.1186/s12889-026-26759-w)
Supplement: Supplementary file 1 — Supplementary Material 1. [file 12889_2026_26759_MOESM1_ESM.docx]

Appendix 1. Search Strategies

| Data bases | Search strategies |
| --- | --- |
| CINAHL: 25 studies | (“human papillomavirus*” OR HPV OR (MH "Human Papillomavirus Viruses")) AND (Video* OR videorecording* OR (MH "Videorecording") OR (MH "Digital Versatile Disc") OR (MH "Videodiscs") OR DVD OR movie* OR film* OR audiovisual* OR (MH "Audiovisuals") OR “audiovisual aids”) |
| PubMed | ("human papillomavirus" OR "human papillomaviruses" OR HPV OR "Human Papillomavirus Viruses"[Mesh]) AND (Video* OR "Videotape Recording"[Mesh] OR "Video Recording"[Mesh] OR DVD OR videodisc* OR movie OR film OR "digital versatile disc" OR "digital versatile discs" OR audiovisual* OR "audiovisual aids" OR "audiovisual aid" OR "Audiovisual Aids"[Mesh]) |
| Scopus | ( TITLE-ABS-KEY ( "human papillomavirus" OR "human papillomaviruses" OR hpv ) AND TITLE-ABS-KEY ( video* OR videorecording* OR dvd OR movie* OR film* OR videodisc* OR "digital versatile disc" OR "digital versatile discs" OR audiovisual* OR "audiovisual aids" ) ) |
| PsycINFO | ("human papillomavirus" OR "human papillomaviruses" OR HPV OR "Human Papillomavirus Viruses"[Mesh]) AND (Video* OR "Videotape Recording"[Mesh] OR "Video Recording"[Mesh] OR DVD OR videodisc* OR movie OR film OR "digital versatile disc" OR "digital versatile discs" OR audiovisual* OR "audiovisual aids" OR "audiovisual aid" OR "Audiovisual Aids"[Mesh]). |
