# Supplementary material for: Video-based interventions to promote HPV vaccination among individuals aged 9 to 26: a systematic review and meta-analysis
Source: BMC Public Health. 2026 Mar 14;26:1308. doi: 10.1186/s12889-026-26759-w (PMC13101323; doi:10.1186/s12889-026-26759-w)
Supplement: Supplementary file 2 — Supplementary Material 2. Table 1. Summary Table. Article 1: “Someone You Love” documentary: using narratives in entertainment media to increase HPV vaccination in Georgia [23]. Article 2: Effects of a narrative HPV vaccination intervention aimed at reaching college women: a randomized controlled trial [31]. Article 3: A storytelling intervention in a mobile, Web-based platform: a pilot randomized controlled trial to evaluate the preliminary effectiveness to promote human Papillomavirus vaccination in Korean American college women [32]. Article 4: 1-2-3 Pap” intervention improves HPV vaccine series completion among Appalachian women [29]. Article 5:How to inform: comparing written and video education interventions to increase human papillomavirus knowledge and vaccination intentions in young adults [18]. Article 6: Predicting Human Papillomavirus Vaccine Uptake in Young Adult Women: Comparing the Health Belief Model and Theory of Planned Behavior [24]. Article 7: Effectiveness of health education programme: Level of knowledge about prevention of cervical cancer among Saudi female healthcare students [38]. Article 8: Survey of girls’ recall of a film providing information on human papillomavirus and cervical cancer 6 months after an offer of vaccination [37]. Article 9: Effects of Educational Interventions on Human Papillomavirus Vaccine Acceptability A Randomized Controlled Trial [33]. Article 10: Effects of information sources in HPV vaccine acceptance: prospective randomized trial [25]. Article 11: The Multidimensional Nature of Perceived Barriers: Global Versus Practical Barriers to HPV Vaccination [34]. Article 12: Designing and Implementing an Educational Social Media Campaign to Increase HPV Vaccine Awareness among Men on a Large College Campus [35]. Article 13: The impact of HPV vaccine narratives on social media: Testing narrative engagement theory with a diverse sample of young adults [28]. Article 14: Using narrative intervention for HPV vaccine behavior c [file 12889_2026_26759_MOESM2_ESM.docx]

Table 1. Summary Table

Article 1: **“Someone You Love” documentary: using narratives in entertainment media to increase HPV vaccination in Georgia**^23^

| **Study** | **Sample Characteristics** | **Study Design** | **Intervention** | **Outcomes** | **Main Results** |
| --- | --- | --- | --- | --- | --- |
| **Title:** Someone You Love” documentary: using narratives in entertainment media to increase HPV vaccination in Georgia.  **Authors:** Darville-Sanders et al (2022)  **Purpose:** To (a) determine the effectiveness of the HPV narrative included in the “Someone You love” documentary on HPV risk perception, vaccine self-efficacy and behavioral intention for HPV vaccine uptake on college students and (b) assess the immediate impact of the documentary on HPV vaccine initiation. | **Region:** Georgia  **Setting:** Research intensive University  Participants: 18~26 years old college students and non-recipients of the HPV vaccine.  **Sample Size:** pre-test: 151 and post-test: 126 students. Only 120 students completed the pre-and-post survey. Only 54 out of 120 completed the follow-up survey.  **Age/grade:** 18–26-year-olds, 40% Freshmen  **Sex:** 76.5% Female and 81.5% heterosexual  **Race:**  58 Caucasian (48.3%)  38 Asian (31.7%)  18 Black or African American (15%)  2 multi-racial (1.7%) | **Design:** Quasi Experimental  **Recruitment:** tabling events, flyers in residence halls and buildings throughout campus, announcements in university classes, email distributions over departmental and college listservs and provided freshmen seminar credits to recruit participants for the documentary screening.  **Incentive:** Participants received a $20 Walmart gift card upon completing the post-survey and an additional $5 gift card upon completing the follow-up survey. | **Duration:** Screen time was for one month  **Theory:** Health Belief Model (HBM) and Transportation Imagery Model (using narrative)  **Program:** Someone You Love: The HPV Epidemic” documentary  **Assessment:** Pre and Post assessment surveys, Follow up Surveys and Qualitative feedback.  Data analysis: dependent samples t-test and ANCOVA. | HPV risk perception, vaccine self- efficacy, intention to get vaccinated.  The follow-up survey consisted of 5 questions. The questions asked if and where the first dose of the HPV vaccine initiated, how many doses were completed after watching the documentary, if the participant intended to complete all 3 doses and if the documentary directly influenced their decision | **Risk Perception:** After watching the documentary movie, the post test scores increased significantly (*p* < .001).  **Self-Efficacy:** Vaccine self-efficacy significantly enhanced after watching the movie (*p* = .04). Meta-analysis: Male: pre: 21.96 ± 7.13 vs post: 23.79 ± 3.79.  Female: pre: 22.98 ± 7.22 vs. post: 23.54 ± 4.29  **Behavioral Intention:** Post-test scores of behavioral intentions increased significantly after watching the movie (*p* < .001) Meta analysis: Male: pre: 15.96 ± 5.56 vs. post: 21.86 ± 6.35 Female: pre: 18.21 ± 5.82 vs. post: 22.88 ± 5.03  **Vaccine uptake:**   1. Post-movie: HPV vaccine was offered after each screening of the documentary. Only 20 students received the first dose of HPV vaccine series. (16%). 2. Follow-up survey: 54 students completed the follow-up survey. 28 out of 54 responded that they have initiated the HPV vaccine.   Qualitative findings: Students provided feedback on “What are some things that you liked about the documentary?” and “In what ways can the documentary improve? What changes do you suggest?” Students’ feedback was positive and supportive.  **Limitations:**   - The study did not employ a control or comparison group, limiting the ability to attribute changes directly to the documentary without considering other external influences. - The use of convenience sampling limits the generalizability of the findings to broader populations outside the study's parameters. - There was a significant dropout rate from pre-test to post-test and post-test to follow-up survey, which may impact the reliability of the findings. - While the study utilized the Health Belief Model (HBM) focusing on public health and behavior change, incorporating communication theories more closely aligned with entertainment-education could enhance understanding of narrative impact. - The study primarily focused on immediate vaccination uptake and intentions shortly after viewing the documentary, lacking in assessment of long-term vaccination completion and behavior change. - The study's results may not fully represent the impacts on diverse demographic groups, including males, LGBTQ+ communities, and racial minorities not adequately represented or focused on in the documentary. |

Article 2: **Effects of a narrative HPV vaccination intervention aimed at reaching college women: a randomized controlled trial**^24^

| **Study** | **Sample Characteristics** | **Study Design** | **Intervention** | **Outcomes** | **Main Results** |
| --- | --- | --- | --- | --- | --- |
| **Title:** Effects of a Narrative HPV Vaccination Intervention Aimed at Reaching College Women: A Randomized Controlled Trial.  **Authors:** Suellen Hopfer (2011)  **Purpose:** Evaluation of a narrative intervention aimed at increasing HPV vaccination among college women. | **Setting**: University health center.  **Participants**: College women aged 18–26 who had not been vaccinated for HPV.  **Sample Size**: 404 female students participated.  **Demographics**: Majority Caucasian (72%), 50% sexually active, 53% had talked with their mother about HPV vaccination. | **Design:** Randomized Controlled Trial (RCT)  **Recruitment**: Participants were college women between the ages of 18 and 26, who had not been vaccinated for HPV. The recruitment was conducted at a university, with 1,000 women randomly sampled from the university health service's database using a random number generator. An email was sent to these women from University Health Services, inviting them to participate in the study. The email included an eligibility statement indicating that only women who had not been vaccinated for HPV were eligible to participate.  **Incentive**: Volunteers who participated in the study and completed the required tasks received a $6 lunch coupon as compensation. | **Theory:** Exemplification theory. Narrative intervention using HPV vaccine decision narratives aimed at increasing HPV vaccination among college women. The intervention was delivered through videos containing narratives from peers, medical experts, or a combination of both.  **Intervention Content:** Developed based on culture-centric narrative theory and exemplification theory, focusing on HPV vaccination narratives. The content included prototypical HPV vaccine decision narratives collected from college women, addressing susceptibility to HPV, vaccine self-efficacy, vaccine safety, and mother-daughter narratives regarding vaccination.  **Format:** The intervention was delivered through videos shown to participants in a computer lab setting. The videos contained narratives either from peers, medical experts, or a combination of both, presenting direct testimonials or re-enacted scenarios to engage the audience.  **Assessment:** Survey Instruments: Pre-intervention and post-test online surveys were administered to collect data on sociodemographic characteristics, sexual activity, HPV knowledge, and communication with parents about HPV vaccination. | **Measures:** The surveys included items to measure HPV vaccination intent, vaccine self-efficacy, and actual uptake of the HPV vaccine 2 months post-intervention.  **Primary Outcome**: Initiation of HPV vaccination.  **Secondary Outcomes:** Vaccine self-efficacy and intent to vaccinate.  Follow-Up: Two months after the intervention, participants were emailed to inquire whether they had received the first dose of the HPV vaccine. | **Peer-only and expert-only narrative interventions** did not significantly increase the odds of vaccination compared to controls. The intervention worked by increasing vaccine self-efficacy and intent to vaccinate.  **Vaccine Uptake:** The intervention resulted in a noticeable increase in vaccine uptake among the participants in the combined peer-expert narrative intervention. The vaccination rates nearly doubled compared to controls (22% vs. 12%). This indicates the effectiveness of the narrative intervention in encouraging vaccine initiation.  **Self-Efficacy:** There was a significant enhancement in vaccine self-efficacy after watching the intervention content. This indicates that the narrative intervention effectively increased participants' confidence in their ability to get vaccinated, addressing potential barriers to vaccination. Meta-analysis: Intervention: 5.95 ± 1.22; Control: 5.7 ± 2.12.  **Intention:**  Meta-analysis: Intervention: 3.10 ± 6.63; Control: 2.81 ± 9.62.  **Risk Perception:** The intervention significantly increased HPV risk perception among the viewers, as indicated by the significant rise in post-test scores. This suggests that the documentary effectively communicated the risks associated with HPV, emphasizing the importance of vaccination for prevention. |

Article 3: **A storytelling intervention in a mobile, Web-based platform: a pilot randomized controlled trial to evaluate the preliminary effectiveness to promote human Papillomavirus vaccination in Korean American college women**^25^

| **Study** | **Sample Characteristics** | **Study Design** | **Intervention** | **Outcomes** | **Main Results** |
| --- | --- | --- | --- | --- | --- |
| **Title:** A storytelling Intervention in a Mobile, Web- Based Platform: A Pilot Randomized Controlled Trial to Evaluate the Preliminary Effectiveness to Promote Human Papillomavirus Vaccination in Korean American College Women.  **Authors:** Minjin Kim, Haeok Lee, Peter Kiang, Teri Aronowitz, Lisa Kennedy Sheldon, Ling Shi, PhD; Jeroan J. Allison (2020)  **Purpose:** (a) To evaluate the preliminary effectiveness of a storytelling video intervention, delivered via a mobile and web-based platform, in promoting Human Papillomavirus (HPV) vaccination among Korean American college women. (b) Address the substantially greater incidence rates of cervical cancer and the lowest rates of cervical cancer screening in this demographic by leveraging culturally relevant storytelling to enhance knowledge, attitudes, and uptake of the HPV vaccine. | **Region: Northeast United States**  **Setting:** Web based, utilizing mobile and online platforms for delivering the intervention and collecting data.  **Participants:** Korean American College Women  **Sample Size:** 104 participants  **Age/grade:** 18 to 26 years  **Sex:** Female  **Race:** Korean American women. | **Design:** Randomized controlled trial  **Recruitment:**  Network-based sampling and convenience sampling.  Bilingual flyers, outreach through social media sites (Facebook, KakaoTalk), and Korean community websites. Leaders of Korean student associations and pastors of Korean churches also helped in distributing study information. The recruitment theme was "I Want to Know More About the HPV Vaccine."  **Incentive**: Individuals who completed the post intervention survey received a $20 gift certificate. Upon completing the 2-month follow-up survey, participants were entered into a raffle with the chance to win one of several gift cards, valued at $10- $100, with multiple winners for each category | **Duration**: Did not specify.  **Theory**: The Situation-specific theoretical framework  **Program**: Experimental group received 17 minute storytelling video intervention featuring stories of Korean American College women’s HPV vaccination experiences.  The comparison group received written information about HPV and the vaccine from authorities. | **Assessment**: Baseline and post intervention surveys measured changes in participants' knowledge, attitudes, and intentions regarding the HPV vaccine.  A follow-up survey conducted two months after the intervention assessed the primary outcome, which was the initiation of HPV vaccination. | **Knowledge about HPV, HPV vaccine, and cervical cancer:** total knowledge scores significantly improved from baseline to postintervention in both groups (*p* < .001). Meta-analysis: Experimental: pre: 12.6 ± 6.4 vs & post: 24.1 ± 4.08  Control: pre:14.4 ± 6.64 vs. post: 23.2 ± 3.93  **Attitude** including cognitive items about the HPV vaccine and the Affective items included direct expression of feelings about getting the HPV vaccine: Both groups had more positive cognitive and affective attitude toward HPV vaccine from baseline to postintervention (< .001); however, there was no group difference Meta-analysis: Affective attitude: Intervention: Pre 11.4 ± 4.46; Post 13.9 ± 4.24; Control: pre 9.6 ± 4.42; post 14.0 ± 3.94  Cognitive attitude: Intervention: Pre 15.0 ± 27.8; Post 38 ± 70.4; Control: pre 21 ± 42; post: 35 ± 70  **Intention to vaccinate:** after the intervention, the intervention group showed 144% increase in intention to receive HPV vaccine while the control group increased by 67%.  **Vaccine uptake:** At the 2-month follow-up, 10 students (22.2%) reported having received at least one dose of the HPV vaccine or having already scheduled the vaccination. The intervention group was twice as likely to report receiving HPV vaccination.  Qualitative findings: Feedback from students about the documentary was overwhelmingly positive, with participants highlighting aspects of the documentary they found impactful. This feedback underscores the potential of storytelling and narrative-based interventions to resonate with young adults and influence their health behaviors.  **Limitations:**   - Reliance on self-reported vaccination status may introduce inaccuracies. - A 2-month follow-up might not capture the long-term effects of the intervention, including the completion of the HPV vaccine series. - The pilot study's small sample size and focus on Korean American college women in the northeast United States restrict the findings' applicability to broader populations. - The study did not employ booster sessions or reminders that could enhance vaccine series completion rates. |

Article 4: **1-2-3 Pap” intervention improves HPV vaccine series completion among Appalachian women^26^**

| **26Study** | **Sample Characteristics** | **Study Design** | **Intervention** | **Outcomes** | **Main Results** |
| --- | --- | --- | --- | --- | --- |
| **Title:** "1-2-3 Pap" Intervention Improves HPV Vaccine Series Completion Among Appalachian  **Authors:** Vanderpool RC, Cohen EL, Crosby RA, Jones MG, Bates W, Casey BR.(2013)  **Purpose:** (a) To evaluate the efficacy of a DVD-based educational intervention, grounded in health behavior theories, aimed at improving the completion rates of the HPV vaccine series among young Appalachian women, a group at high risk for cervical cancer but with low HPV vaccination rates. (b) Identify correlates of intent to complete the vaccine series, actual series completion, and test the effectiveness of the intervention in a community setting, highlighting the intervention's potential to enhance health equity and reduce cervical cancer disparities in medically underserved regions. | **Region: Appalachian Kentucky, United States.**  **Setting:** Eight county catchment area of Appalachian Kentucky, USA.  **Participants:** Young women residing in the specified Appalachian region  **Sample Size:** 344 women  **Age/grade:** 18-26 years old  **Sex:** Female  **Race:** 94% non- Hispanic white | **Design:** Randomized Controlled Trial  Participants were randomized into two groups: one received the DVD intervention plus standard-of-care (including follow-up telephone reminders for doses 2 and 3), and the other received only the standard-of-care including an informational HPV vaccine brochure.  **Recruitment:**  A social marketing campaign informed by Rogers’ diffusion of innovations theory  **Incentive:**   - $25 gift card - Free t-shirt | **Duration**: Did not specify; however, medical record review for up to nine months past the initial vaccine dose.  **Theory**: Information, Motivation, Behavioral Skills model (IMB)  **Program**: 13-minute educational DVD titled "1-2-3 Pap." The DVD content, guided by formative research and the IMB model  **Assessment**:  Intention to complete the three-dose vaccine series as well as actual series completion. | 1. Positive intent to complete the vaccine series emerged as a strong predictor of actual series completion. | **Intention to Vaccinate:** Positive intent to complete the vaccine series was indicated by 64.3% of the women (n = 220).  **Vaccine uptake:** One-third of participants with positive intent completed the three doses. Women assigned to the intervention were 2.44 times more likely than women in the control group to complete the series.  **Limitations:**   - Did not include a probability sample of patients. Did not measure other variables such as the influence of subjective norms, perceived behavioral control, and behavioral intention. |



Article 5: **How to inform: comparing written and video education interventions to increase human papillomavirus knowledge and vaccination intentions in young adults**^18^

| **Study** | **Sample Characteristics** | **Study Design** | **Intervention** | **Outcomes** | **Main Results** |
| --- | --- | --- | --- | --- | --- |
| **Title:** How to Inform: Comparing Written and Video Education Interventions to Increase Human Papillomavirus Knowledge and Vaccination Intentions in Young Adults  **Authors:** Andrea Krawczyk, Elsa Lau, Samara Perez, Vanessa Delisle, Rhonda Amsel, Zeev Rosberger (2012)^18^  **Purpose:** The purpose of the study was (a) To evaluate the relative efficacy of written and video educational interventions in increasing knowledge about Human Papillomavirus (HPV) and the HPV vaccine, as well as intentions to receive the HPV vaccine among college students. It also aimed (b) To determine if the format of the intervention (written vs. video) influenced the effectiveness of the educational content in improving HPV knowledge and vaccination intentions. | **Region: Montreal, Quebec, Canada.**  **Setting**: A university in Montreal, providing a controlled environment for the intervention and assessment.  Participants: Male and female undergraduates.  **Sample Size**: 200 undergraduates.  **Age/grade**: Undergraduate students  **Sex**: 60 male and 140 female.  **Race**: White (Caucasian) participants (61%)  Other ethnic groups making up the remainder. | **Design:**  Randomized Controlled Trial  **Recruitment:** Advertisements in university classes  **Incentive:** Course credit or were entered into a draw for a chance to win one of three $100 prizes. | **Duration**: Did not specify.  **Theory**: Health Belief Model (HBM)  **Program**: The educational content for both the written and video interventions was derived from authoritative health websites and was identical in information but varied by delivery method. The video intervention featured a healthcare professional speaking directly to the audience.  **Assessment**: Pre- and post-intervention questionnaires were used to measure changes in HPV knowledge and vaccination intentions, utilizing a mix of specific knowledge questions and intention scales | 1. Knowledge about cervical cancer, HPV, and the HPV vaccine,    1. Intentions to receive the HPV vaccine, and    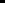 | **Knowledge:** Knowledge significantly increased in both the written and video intervention groups (p < .001), with no improvement observed in the control group. There was no significant difference in knowledge gains between the written and video groups.  Meta-analysis: Video n = 74, pre: 10.89 ± 4.5, post: 12.06 ± 4.15  Written n =61, pre: 10.48 ± 4.86, post: 17.46 ± 2.09  Control n = 65, pre: 11.49 ± 4.25, post: 16.7 ± 2.19  **Intention:** Intention to receive the HPV vaccine significantly increased in the written intervention and video intervention groups (*p* < .001).  Meta-analysis: Video intervention n = 61: pre 3.14 ± 1.85; post 4.39 ± 1.86;  Written intervention n = 74: pre: 3.52 ± 1.94; post: 4.57 ± 1.90;  Control n = 65: pre 3.51 ± 1.90; post: 3.88 ± 1.77  **Limitations:**   - Results can’t be generalizable to a wider population of young adults. Participants were self-selected, which may have also limited generalizability as the sample may be representative of individuals who are interested in health and sexual health. - Did not measure long-term effects of the intervention on knowledge and intention. |

Article 6: **Predicting Human Papillomavirus Vaccine Uptake in Young Adult Women: Comparing the Health Belief Model and Theory of Planned Behavior^27^**

| **Study** | **Sample Characteristics** | **Study Design** | **Intervention** | **Outcomes** | **Main Results** |
| --- | --- | --- | --- | --- | --- |
| **Title:** Predicting Human Papillomavirus Vaccine Uptake in Young Adult Women: Comparing the Health Belief Model and Theory of Planned Behaviour  **Authors:** Mary A. Gerend, Janet E. Shepherd (2012)  **Purpose:** To compare the predictive ability of the HBM and TPB in predicting HPV vaccination among young adult women | **Region:** Southeastern United States  **Setting**: Large University  **Participants:** 18-26 years old Female college students  **Sample Size**: 739 participants completed the survey; HPV vaccine uptake assessed 10 months later.   1. Gain-framed (n=205) 2. Loss-framed (n=243) 3. Control (n=246)   **Age/grade**: 18-26 years, Mean age=21 years.  **Sex**: Female  **Race**: 16% Hispanic or Latina, 68% white, 19% black or African American | **Design:**  Randomized Clinical Trial  **Recruitment:** Flyers, online announcements on university’s course management system and Department of Psychology subject pool.  **Incentive:** $30 for general student body participants and course credit for Department of Psychology recruits. | **Duration**:  Baseline data collection from March to August 2009 with a follow up approximately 10 months later from January to June 2010.  **Theory**: Health Belief Model (HBM) and Theory of Planned Behavior (TPB)  **Program**: Educational videos on HPV vaccination.   1. The gain-framed video emphasized the benefits of getting vaccinated 2. The loss-framed video emphasized the costs of not getting vaccinated 3. Participants in the control group received no framed information.   **Assessment**: Survey on HBM and TPB constructs; HPV vaccine uptake assessed 10 months post-intervention  **Data analysis:** Structural equation modeling (SEM), Health Belief Model (HBM) and Theory of Planned Behaviour (TPB). | 1. Health Belief Model constructs including perceived susceptibility to and perceived severity of genital HPV infection, cervical cancer, and genital wart, perceived benefits of HPV vaccination, and perceived barriers to HPV vaccination; 2. Theory of Planned Behavior constructs including attitude, subjective norms, and perceived behavioral control were assessed with multiple items. 3. Uptake of the HPV vaccine, assessed 10 months after the initial intervention through self-reported surveys. | **Health Belief Model construct:** Significant predictors of uptake included perceived susceptibility, safety concerns, vaccine cost, and physician recommendation. Perceived severity and perceived benefits were not associated with uptake.  Meta-analysis: Beliefs: r = 0.36, p<.01  **Theory of Planned Behavior construct**: Attitude, subjective norms, and self-efficacy were all positively related to intention, and intentions were positively related to vaccine uptake.  **Vaccine uptake:** As of the 10-month follow-up, rate of HPV vaccine uptake were equivalent across the 3 groups: 12 participants in the gain-framed group, 14 participants in the loss-framed group, and 15 participants in the control group received HPV vaccine.  **Limitations:**   1. Using self-efficacy as a marker for perceived behavioral control. Self-efficacy captured only one aspect of perceived behavioral control. 2. Results can’t be generalizable to other populations. |

Article 7: **Effectiveness of health education programme: Level of knowledge about prevention of cervical cancer among Saudi female healthcare students**^35^

| **Study** | **Sample Characteristics** | **Study Design** | **Intervention** | **Outcomes** | **Main Results** |
| --- | --- | --- | --- | --- | --- |
| **Title:** Effectiveness of Health Education Programme: Level of Knowledge about Prevention of Cervical Cancer among Saudi Female Healthcare Students  **Authors:** Ghadeer Khaled Al-Shaikh, Sadiqa Badar Syed, Amel Ahmed Fayed, Reem Ali Al-Shaikh, Eman Mohammed Al-Mussaed, Farida Habib Khan, Hala Elmorshedy (2017)  **Purpose:** To (a) evaluate the effectiveness of a health education programme on the knowledge of human papillomavirus (HPV) among female medical students at Princess Nourah bint Abdulrahman University, Riyadh, Saudi Arabia. The programme aimed to (b) increase awareness about cervical cancer prevention, HPV risk factors, and the importance of HPV vaccination through lectures, videos, and posters. | **Region:** Riyadh, Saudi Arabia  **Setting:** Princess Nourah bint Adbulrahman University  **Participants:** Female healthcare students  **Sample Size:** 535 undergraduate students  **Age/grade:** Mean age of 20.3 ± 1.3 years  **Sex:** Female  **Race:** Not specified explicitly. | **Design:** Quasi-experimental.  **Recruitment:** Non-probability convenience sampling technique; students who gave consent to participate and filled in the questionnaire completely were included.  **Incentive:** Certificate of participation and symbolic prizes for volunteers | **Duration:** Two weeks for the intervention program.  **Theory:** Health education theories aimed at improving knowledge and behavior regarding cervical cancer prevention. (No models mentioned explicitly)  **Program:** Multidirectional unified intervention program including lectures, videos, posters, peer education, and passive health education messages. Covered risk factors of cervical cancer, clinical presentation, oncogenic strains of HPV, and detailed information about HPV vaccines and Pap smear. | 1. Knowledge on cervical cancer, 2. Knowledge about HPV on risk factors, 3. Knowledge about Pap smear 4. Knowledge on HPV vaccine | Knowledge about HPV increased significantly (p < .001). A significantly higher percentage of students were able to define HPV and its risk factors following the intervention. Additionally, knowledge regarding sensitivity and time to perform Pap smear testing showed a significant improvement.  **Limitations:**   1. The study was conducted in one university and included only female students. Unequal participation from different health colleges, with only first and second-year students included from the medical and nursing colleges. 2. Potential for confounding variables, as clinical subjects start from the third year for medical and nursing students. 3. Limited generalizability due to the specific demographic and geographic focus |

Article 8: **Survey of girls’ recall of a film providing information on human papillomavirus and cervical cancer 6 months after an offer of vaccination**^34^

| **Study** | **Sample Characteristics** | **Study Design** | **Intervention** | **Outcomes** | **Main Results** |
| --- | --- | --- | --- | --- | --- |
| **Title:** Survey of Girls’ Recall of a Film Providing Information on Human Papillomavirus and Cervical Cancer 6 Months After an Offer of Vaccination  **Authors:** Loretta Brabin,  Rebecca Stretch, Stephen Roberts, Peter Elton,  David Baxter,  Rosemary McCann (2010)  **Purpose:** The purpose of this study was to evaluate the recall of information about human papillomavirus (HPV) and cervical cancer among pre-adolescent girls approximately six months after they had been offered the HPV vaccine and viewed an educational film. The study aimed to assess whether the film influenced their decision to get vaccinated and what key information they retained over time. | **Region:** Manchester, United Kingdom  **Setting:** Secondary schools within the jurisdiction of two Primary Care Trusts in Manchester  Participants: Pre-adolescent girls who were offered the HPV vaccine  **Sample Size:** 38% (n=1084) of 2,853 eligible parents agreed to be contacted. 553 girls returned a questionnaire and 63% of them had seen the film.  **Age/grade:** 12-13 years old / Year 8  **Sex:** Female  **Race:** Not specified in the study | **Design:** Quasi-experimental design  **Recruitment:** Parents of eligible girls were contacted twice to provide consent for their daughters to participate in follow-up research questionnaires. Those who agreed (1084 parents) were asked to pass on the survey to their daughters to complete and return in a pre-stamped, addressed envelope.  **Incentive:** Not specified in the study | **Duration:** The survey assessed recall details of the film (knowledge) on HPV and cervical cancer approximately 6 months after the girls had been offered the HPV vaccination.  **Theory:** The study was guided by the Elaboration Likelihood Model, which focuses on how messages are received and processed, influencing message retention and attitudes.  **Program:** The program involved showing an educational film titled "HPV, what’s that Miss?" to pre-adolescent girls to inform them about HPV and cervical cancer, as part of the Manchester HPV vaccine study. | 1. This study focuses specifically on the impact of the film on the girls’ Recall of Film Content. 2. Influence on Vaccination Decision 3. Attitudes Toward Vaccination 4. Knowledge of HPV and Cervical Cancer. | **Recall of Information:** Of 346 girls who had seen the film, only 42% of the girls attempted the questionnaires. Among them, 60% (n=88) gave correct answers about the film’s actual content. However, the majority could not recall any key messages from the film after six months.  **Vaccination Decision:** Girls who viewed the film were more likely to report having received enough information (88% vs 76%) and to have wanted the vaccine (90% vs 83%) compared to those who did not view the film.  **Attitudes:** Girls who saw the film were significantly more aware of the risks of future sexual relationships and were less reluctant to discuss the vaccine with a future boyfriend. They also expressed more positive attitudes toward the importance of vaccination for their health.  **Limitations:**   1. Sample Bias, Variation in Film Exposure: Differences in film viewing between schools and potential absenteeism during the film sessions may have influenced who saw the film and thus their recall and attitudes; Recall Challenges: Asking girls to recall facts rather than providing true/false or multiple-choice questions may have underestimated their knowledge due to the difficulty of the task. 2. Cross-Sectional Design: The study's cross-sectional design limits the ability to establish causality between viewing the film and changes in knowledge or attitudes; and Parental Influence: Although there was little evidence of parental assistance with the questionnaire, parental influence on the girls' responses cannot be entirely ruled out. |

Article 9: **Effects of Educational Interventions on Human Papillomavirus Vaccine Acceptability A Randomized Controlled Trial^28^**

| **Study** | **Sample Characteristics** | **Study Design** | **Intervention** | **Outcomes** | **Main Results** |
| --- | --- | --- | --- | --- | --- |
| **Title:** Effects of Educational Interventions on Human Papillomavirus Vaccine Acceptability: A Randomized Controlled Trial  **Authors:** Lori Cory, Susan Ellenberg, Hillary R. Bogner,  Wei-Ting Hwang, Jennifer S. Smith, Ashley Haggerty, Mark Morgan, Robert Burger, Christina Chu, Emily Ko (2019)  Purpose: The purpose of this study was to estimate whether targeted educational interventions could increase human papillomavirus (HPV) vaccine acceptability and knowledge among young women. The study aimed to determine the effectiveness of different educational approaches (handouts and videos) in improving vaccine acceptance and understanding of the HPV vaccine's benefits and risks. | **Region:** Philadelphia, Pennsylvania, USA  **Setting:** Obstetrics and Gynecology clinics affiliated with the University of Pennsylvania Healthcare System  Participants: Young women presenting for gynecologic or obstetric care  **Sample Size:** 256 women  **Age/grade:** 12-26 years old  **Sex:** Female  Race: Predominantly Black (80.6%)  Other races include American Indian or Alaskan Native, Asian, Native Hawaiian or other Pacific Islander, White, and other races | **Design:** Randomized controlled trial with an exploratory phase  Recruitment: Participants were recruited from Obstetrics and Gynecology clinics affiliated with the University of Pennsylvania Healthcare System. Eligible women were approached in the clinic for recruitment and those who provided written consent were randomized into one of three study arms: control (no educational intervention), educational handout, or educational video.  Incentive: A $20 gift card stipend was provided to participants who completed the semi-structured qualitative interviews during the exploratory phase of the study. | **Duration:**  The exploratory phase took place from May 2013 to January 2014 and the randomized controlled trial phase occurred from March 2017 to August 2017.  **Theory:** Data from the exploratory study were used to identify themes driving acceptance nonacceptance.  **Intervention**: The program included two educational interventions:  Educational Handout: A one-page informational sheet and  Educational Video: An approximately eight-minute video. Both interventions provided information on HPV, its link to cervical cancer, and the benefits of the HPV vaccine, targeting knowledge deficits identified in the exploratory phase.  **Assessment:**  Participants completed a survey collecting data on demographics, HPV vaccine preferences, and HPV vaccine knowledge after completing their assigned intervention. The primary outcome was HPV vaccine acceptability, and the secondary outcome was HPV vaccine knowledge.  **Data analysis:** | 1. HPV Vaccine Acceptability 2. HPV Vaccine Knowledge   Utility of Educational Interventions: The perceived helpfulness of the educational handout and video in learning about the HPV vaccine and making a decision about vaccination.  Key Findings:  HPV Vaccine Acceptability:  51.7% of participants in the educational video arm reported willingness to accept the HPV vaccine.  33.3% of participants in the educational handout arm reported willingness to accept the HPV vaccine.  28.2% of participants in the control arm reported willingness to accept the HPV vaccine.  The differences in acceptability rates were statistically significant (P < 0.01).  HPV Vaccine Knowledge:  Median knowledge scores were higher in the educational video arm (6 out of 8) compared to the educational handout arm (5 out of 8) and the control arm (3 out of 8) (P < 0.01).  Participants in the educational video arm were more likely to respond correctly to all eight knowledge statements.  Utility of Educational Interventions:  97.7% of participants in the educational video arm found the intervention helpful in learning about the HPV vaccine, compared to 92.9% in the educational handout arm (P = 0.15).  86.2% of participants in the educational video arm found the intervention helpful in deciding whether to accept the HPV vaccine, compared to 70.2% in the educational handout arm (P < 0.01). | **Vaccine uptake:**   1. In the exploratory study, 51% (n=102) of women reported willingness to accept the HPV vaccine. Despite this, 41% (n=82) and 19% (n=38) of women initiated and completed the vaccine series. 2. In the RCT study, 52% (n=45) of women in the educational video reported the highest vaccine acceptability rates compared with 33% (n=28) in the educational handout group (*p* < .001).   **HPV vaccine knowledge:**   1. In the exploratory study, vaccine acceptors were more likely than nonacceptors to correctly understand the role of the HPV vaccine in preventing cervical cancer (51% Vs 22%, *p* < .001). They were also more understand the role for ongoing cervical cancer surveillance with the Pap test (73% vs 54%), the route of spread of HPV (49% vs 39%), and the ability of obtain the HPV vaccine after sexual debut (87% vs 71%) when compared with nonacceptors. 2. In the RCT study, women assigned to the video and handout groups had significantly higher knowledge score when compared with the control group (*p* < .001). There was a significant difference on the HPV vaccine prevents cervical cancer among three groups. More women answered this question correctly in the video and handout groups when compared with the control group (*p* < .001).   **Limitations:**   1. Selection Bias; Generalizability; Baseline Acceptance Differences: Differences in baseline acceptance rates between the exploratory phase and the randomized controlled trial phase could be due to national and local campaigns aimed at increasing vaccine uptake; 2. Single Institution: Conducting the study at a single institution may limit the ability to generalize findings to other settings or populations; 3. Intervention Delivery: Variability in how the educational interventions were delivered and received could influence the outcomes. |

Article 10: **Effects of information sources in HPV vaccine acceptance: prospective randomized trial^36^**

| **Study** | **Sample Characteristics** | **Study Design** | **Intervention** | **Outcomes** | **Main Results** |
| --- | --- | --- | --- | --- | --- |
| **Title:** Effects of Information Sources in HPV Vaccine Acceptance: Prospective Randomized Trial  **Authors:**  Fırat Ekmez & Murat Ekmez (2022)  **Purpose:** To evaluate the impact of different information sources (verbal, written, and visual) provided by healthcare workers on the acceptance of the HPV vaccine among women. The study aimed to determine which method of information delivery is most effective in reducing anxiety and increasing vaccine acceptance | **Region:** Silopi and Istanbul, Turkey  **Setting:** Department of Gynecology and Obstetrics  Participants: Women aged 18-26 years old attending a gynecology outpatient polyclinic  **Sample Size:** 225 participants (75 per group)  **Age/grade:** 18-26 years old  **Sex:** Female  **Race:** Not specified in the study | **Design:** Randomized controlled trial  Recruitment: Participants were recruited from the gynecology outpatient polyclinic. They were randomly assigned to one of three groups using a computer-assisted randomization program.  Incentive: Not specified in the study | Duration: The study was conducted between September 2020 and June 2021.  **Theory:** The study was based on the premise that providing comprehensive and accessible information through different mediums can influence health behaviors, such as vaccine acceptance, by addressing knowledge gaps and reducing anxiety.  **Intervention**: Participants were divided into three groups:  Group 1: Received a 20-minute informative talk by professional healthcare providers about the benefits of HPV vaccination.  Group 2: Received the same informative talk supplemented with a 1200-word, three-page written information source about the HPV vaccine.  Group 3: Received the same informative talk supplemented with a five-minute video about the HPV vaccine. | 1. HPV Vaccine Acceptance 2. Anxiety | **HPV Vaccine Acceptance:** The differences in acceptance rates between groups were statistically significant (P = 0.001).   1. Group 1 (Verbal Information): Acceptance rate increased from 26.7% to 56.0%. 2. Group 2 (Verbal + Written Information): Acceptance rate increased from 24.0% to 58.7%. 3. Group 3 (Verbal + Video Information): Acceptance rate increased from 28.0% to 82.7%.   **Beck Anxiety Inventory:**  After the intervention, all three groups showed a decrease in anxiety score, but this decrease was significantly greater for participants receiving information from professional health-care providers supplemented by video content (*p* = .001).  **Limitations:**   1. Single-Site Study 2. Exclusion of Younger Age Group: The study did not include participants under 18, who are a significant target group for HPV vaccination. 3. Potential Bias: Participants' responses could be influenced by internal (mental status, physical fatigue) and external factors, although efforts were made to minimize these by providing a comfortable environment. 4. Short Follow-Up: The study only assessed outcomes one week after the intervention, not capturing long-term effects on vaccine uptake and sustained behavior change. |

Article 11: **The Multidimensional Nature of Perceived Barriers: Global Versus Practical Barriers to HPV Vaccination**^29^

| **Study** | **Sample Characteristics** | **Study Design** | **Intervention** | **Outcomes** | **Main Results** |
| --- | --- | --- | --- | --- | --- |
| **Title:** The Multidimensional Nature of Perceived Barriers: Global Versus Practical Barriers to HPV Vaccination  **Authors:** Mary A. Gerend, Melissa A. Shepherd, Janet E. Shepherd (2013)  **Purpose:** To examine the multidimensional nature of perceived barriers to health behavior, specifically HPV vaccination, and to determine how these barriers vary between individuals intending to engage in the behavior and those not intending to engage. | **Region:** Southeastern United States  **Setting:** Florida State University, including the general student body and the psychology department subject pool.  **Participants:** Young adult women aged 18–26 who had not received any doses of the HPV vaccine and were not currently pregnant.  **Sample Size:** 703 participants completed the study out of the initial 739 who were enrolled.  **Age/grade:** Participants were aged 18–26, with a mean age of 21 years.  **Sex:** Female  **Race:** The majority of participants self-identified as White (68%) or Black/African American (19%). Other participants identified as multiracial, Asian/Asian American, Native Hawaiian/Pacific Islander, or American Indian/Alaska Native | **Design:** Quasi-experimental design  **Recruitment:** Participants were recruited via flyers, announcements on the university's web-based course management system, and the psychology department subject pool.  **Incentive:** Participants received $30 for completing the baseline session and a $10 gift card for completing the follow-up survey. Psychology department participants received course credit instead of monetary compensation. | **Duration:** Did not specify.  **Theory:** Health belief model (HBM)  **Program:** Participants viewed an educational video about HPV vaccination and completed surveys assessing their perceived barriers and intentions to receive the vaccine.  **Assessment:** Participants reported their perceived barriers to HPV vaccination and intentions to receive the vaccine two months after the initial educational intervention. | 1. Behavioral Intentions 2. Perceived Barriers to HPV Vaccination 3. Vaccine Uptake | **HPV vaccine uptake:** Two months after the intervention, 17 participants reported that they had received at least one dose of the HPV vaccine.  **HPV vaccine intentions:** Two months after the intervention, 27% (n=187) indicated that they were planning to get vaccinated for HPV sometimes in the future, 30% (n = 209) indicated that they were not planning to get vaccinated, and 44% (n = 307) were undecided.  **Perceived barriers to HPV vaccination:** Intenders’ concerns about vaccine safety were significantly lower than both nonintenders and undecided participants. Mean levels for the perceived lack of need composite also clearly demarcated the three intentions groups (*p* < .001). Concerns about vaccine expense were stronger for both intenders and undecided participants relative to nonintenders (*p* < .001).  **Limitations:**   1. The follow-up period for the study was two months. Future research may benefit from a longer-term, longitudinal assessment of perceived barriers, as well as the use of a specific stage-based model. 2. The current study measured intentions rather than actual behavior. Future research should explore whether perceived barriers at a given point in time can predict who ultimately adopts or does not adopt the behavior. |

Article 12: **Designing and Implementing an Educational Social Media Campaign to Increase HPV Vaccine Awareness among Men on a Large College Campus^30^**

| **Study** | **Sample Characteristics** | **Study Design** | **Intervention** | **Outcomes** | **Main Results** |
| --- | --- | --- | --- | --- | --- |
| **Title:** Designing and Implementing an Educational Social Media Campaign to Increase HPV Vaccine Awareness among Men on a Large College Campus  **Authors:** Connor T. Hughes, Susan Kirtz, Lois M. Ramondetta, Qian Lu, Dalnim Cho, Charlotte Katzin, Lee Ann Kahlor (2020)  **Purpose:** The purpose was to identify predictors of self-reported HPV vaccine uptake and to target these factors in a health education campaign aimed at male students on a large college campus. | **Region:** The University of Texas at Austin  **Setting:** Large public university campus  **Participants:** Male students at the university who were English-speaking, aged 18-26, and currently enrolled.  **Sample Size:** 90 eligible males completed the pre-campaign survey; 41 of these participants completed both pre- and post-campaign surveys.  **Age/grade:** Participants were aged 18-26, with a mix of undergraduates and possibly some graduate students.  **Sex:** Male  **Race:** Ethnicity of respondents closely reflected the overall university demographics: Non-Hispanic White (48%), Hispanic (23%), Black or African American (4%), Asian or Asian American (18%), and other (7%) | **Design:** Quasi-experimental study using observational cohort study with pre- and post-campaign surveys  **Recruitment:** Participants were recruited via email lists from student organizations and classes, including organizations of various ethnicities, genders, and interests.  **Incentive:** Did not specified in the provided text. | **Duration:** The campaign was conducted over four weeks, from March to April 2016.  **Theory:** The campaign was based on the Health Belief Model (HBM) and Theory of Planned Behavior (TPB), targeting health belief constructs associated with vaccine uptake.  **Program:**  Pre- and post-campaign surveys assessing health beliefs and information-seeking behavior.  Development and distribution of educational materials based on survey results.  Social media marketing through the university health center's Facebook page.  A series of three online videos addressing different aspects of HPV and the vaccine.  In-person presentations to student organizations.  Articles in the campus newspaper and digital posters in high-traffic areas. | 1. Health belief related to perceived susceptibility 2. Health belief related to perceived severity 3. Health belief related to self-efficacy 4. Health belief related to subjective norms 5. Health belief related to barriers 6. HPV vaccination uptake at the university health clinic 7. HPV information seeking behavior   Significant improvement in health beliefs related to perceived susceptibility (p < .01), perceived severity (p < .05), and self-efficacy (p < .01).  Increase in information-seeking behavior via social media, flyers, internet videos, and friends.  Significant increase in HPV vaccination uptake at the university health clinic during the campaign period. | **Health belief related to perceived susceptibility:** significant improvement (*p* < .01).  **Health belief related to perceived severity:** significant improvement (*p* < .005).  **Health belief related to self-efficacy:** significant improvement (*p* < .01).  **Health belief related to subjective norms and barriers:** slightly improved.  **HPV information seeking behavior:** there was a significant change in the HPV information available to males between pre-and post-intervention  **Vaccine uptake:** fifteen male students received vaccine before and another 35 male students received the HPV vaccine after the campaign.  **Limitations:**   1. Sample Size and Generalizability: Small sample size and convenience sampling limit generalizability. 2. Students were not randomly assigned and no control groups. 3. Response Rate: Low response rate and difficulty in contacting subjects for follow-up. |

Article 13: **The impact of HPV vaccine narratives on social media: Testing narrative engagement theory with a diverse sample of young adults^31^**

| **Study** | **Sample Characteristics** | **Study Design** | **Intervention** | **Outcomes** | **Main Results** |
| --- | --- | --- | --- | --- | --- |
| **Title:** The impact of HPV vaccine narratives on social media: Testing narrative engagement theory with a diverse sample of young adults  **Authors**: Amy E. Leader, Michelle Miller-Day, Rikishi T. Rey, Preethi Selvan, Anne E. Pezalla, Michael L. Hecht (2022)  **Purpose**: To test the impact of a social media campaign with narrative-based health information on intentions related to HPV vaccination among young adults | **Region**: United States  **Setting**: Social media (Facebook, Instagram  **Participants:** Young women aged 18-26 years  **Sample Size:** 991 completed surveys, 607 usable surveys  **Age/Grade:** 95% aged 18-26  **Sex:** Female  **Race:**  37% White/Caucasian,  26% Asian/Asian American, 15% Hispanic/Latino/Latina, 10% Black/African American, 6% More than one ethnicity, 6% Other | **Design:** Quasi-experimental design  **Recruitment:** Sponsored ad campaign on Facebook targeting young women (18-26 years)  **Incentive:** $10 electronic gift card for completing the survey | **Duration:** The study ran from August 12, 2020 through September 26, 2020.  **Theory:** Narrative Engagement Theory  **Program:** Six narrative-based videos focusing on HPV vaccination  **Assessment:** Survey post-video on engagement, intentions to talk about HPV vaccine, and intentions to get vaccinated  **Data Analysis:** Pearson correlation, ANOVA, moderation analysis using PROCESS macro in SPSS | 1. Engagement: the engagement variable measurement assessed interest, realism, and identification. 2. Intentions to talk with a healthcare provider or their friends or family about the vaccine or to receive the HPV vaccine the next time when they visit their doctor. | **Engagement:** Higher engagement with the videos was positively correlated with stronger intentions to talk to a healthcare provider (r = 0.44, p = .01), talk to friends or family (r = 0.52, p = .01), and get vaccinated against HPV (r = 0.43, p = .01).  **Intentions:** The highest intention score was for talking to a healthcare professional (M = 3.89, SD = 1.02), followed by getting the HPV vaccine (M = 3.71, SD = 1.08), and talking to friends or family (M = 3.65, SD = 1.12).  The study demonstrated that narrative-based videos on social media could effectively engage young adults and positively influence their intentions regarding HPV vaccination. The results support the predictions of Narrative Engagement Theory, which predicts that engaging narratives can enhance the relevance and impact of health messages.  **Limitations:** Inability to measure actual HPV vaccination behavior, potential social desirability bias, findings not generalizable to other social media platforms or young men. |

Article 14: **Using narrative intervention for HPV vaccine behavior change among Khmer mothers and daughters: A pilot RCT to examine feasibility, acceptability, and preliminary effectiveness^32^**

| **Study** | **Sample Characteristics** | **Study Design** | **Intervention** | **Outcomes** | **Main Results** |
| --- | --- | --- | --- | --- | --- |
| **Title:** Using narrative intervention for HPV vaccine behavior change among Khmer mothers and daughters: A pilot RCT to examine feasibility, acceptability, and preliminary effectiveness  **Authors**: Haeok Lee, Minjin Kim, Mary E. Cooley, Peter Nien-chu Kiang, Deogwoon Kim, Shirley Tang, Ling Shi, Linda Thiem, Penhsamnang Kan, Sonith Peou, Chhan Touch, Phala Chea, Jeroan Allison (2018)  **Purpose**: To develop a theory-guided culturally grounded narrative intervention to promote HPV vaccination behavior and examine the feasibility, acceptability, and preliminary effectiveness of the intervention among dyads of Cambodian American mothers and daughters | **Region**: Massachusetts, United States  **Setting**: Community health centers, homes, and Khmer community centers  **Participants:** Khmer American mothers and their daughters aged 14-17 years  **Sample Size**: 19 dyads of Khmer mothers and daughters (38 participants total)  **Age/Grad**e: Daughters aged 14-17 years, mothers' ages not specified  **Sex**: Female  **Race**: Khmer American | **Design**: Pilot randomized controlled trial (RCT)  **Recruitment**: Community-based participatory research methods, including recruitment through community health centers, Khmer community organizations, and local media.  **Incentive**: $50 gift card for each participant after completing the post-test | **Duration**: Recruitment occurred over 7 months, with follow-up assessments at 1 month post-intervention  **Theory**: Revised Network Episode Model (rNEM) and Storytelling Narrative Communication theory  **Program**: Development of storytelling narrative videos titled “Save My Daughter from Cervical Cancer: Stories by Khmer Mothers and Daughters”, presented in both Khmer and English. These videos were presented to the participants. All participants in the intervention group watched the entire storytelling video, which lasted approximately 28 minutes. Control group participants read the written materials, which took about 10 minutes. | 1. Intentions to vaccination 2. Knowledge about HPV and HPV vaccine 3. Attitude toward HPV vaccine   Conclusion: The study demonstrated the feasibility and acceptability of using a narrative-based intervention to promote HPV vaccination among Khmer mothers and daughters. The findings suggest that culturally tailored storytelling can effectively engage and educate participants, leading to increased intention to vaccinate. The study highlighted significant communication barriers between mothers and daughters, primarily due to language differences.  The positive outcomes and feedback from this pilot RCT support the feasibility and potential effectiveness of narrative interventions for improving HPV vaccination behavior among American populations. | **Intentions to Vaccinate**: Daughters in the intervention group reported a higher intention to receive the HPV vaccine within one month compared to the control group (4 vs. 1). However, actual vaccine initiation was the same in both groups (2 vs. 2).  **Knowledge and Attitudes:** Although the study did not measure long-term outcomes, immediate post-intervention assessments showed increased knowledge and positive attitudes toward HPV vaccination among participants in the intervention group.  **Qualitative Findings**: Participants reported high acceptability of the narrative intervention. The relatable, culturally relevant stories and familiar characters enhanced engagement and emotional connection. Many noted the videos positively influenced their attitudes toward HPV vaccination.  **Limitations**: Small sample size, inability to measure actual vaccine uptake beyond initial intention, potential bias due to self-reporting, and challenges in recruiting and retaining dyads of mothers and daughters. The study did not measure long-term outcomes |

Article 15: **Raising cervical cancer awareness: Analysing the incremental efficacy of Short Message Service (SMS) combined with video intervention^37^**

| **Study** | **Sample Characteristics** | **Study Design** | **Intervention** | **Outcomes** | **Main Results** |
| --- | --- | --- | --- | --- | --- |
| **Title:** Raising cervical cancer awareness: Analysing the incremental efficacy of Short Message Service (SMS) combined with video intervention  **Authors**: Marina Serra Lemos, Inês Areal Rothes, Filipa Oliveira, Luisa Soares (2017)  **Purpose**: To evaluate whether combining SMS with a brief video enhances the effectiveness of a cervical cancer prevention education intervention—beyond the video alone—by improving key determinants of health behavior change: knowledge, motivation, and intention. | **Region**: Portugal  **Setting**: University of Madeira, Portugal  **Participants:** Portuguese female college students  **Sample Size**: 144 participants divided into three groups: Control (31), Video-only (82), SMS + Video (31)  **Age/Grade**: Mean age of 21.13 years (SD = 3.19)  **Sex**: Female  **Race**: Not specified | **Design**: Quasi-experimental  **Recruitment**: Recruited from various undergraduate courses, volunteers.  **Incentive**: Not specified | **Duration**: Six weeks (SMS intervention lasted five weeks)  **Theory**: Social Cognitive Theory (SCT), Theory of Planned Behavior (TPB)  **Program**:  Video Intervention: 12-minute educational video on cervical cancer prevention  SMS Intervention: Five weekly SMS messages containing information, tips, and myth/fact format about cervical cancer prevention  **Assessment**: Pre- and post-intervention measures using the Cervical Cancer Questionnaire (CCQ), assessing knowledge, self-efficacy, outcome expectations, and intentions | 1. Knowledge regarding potential risk factors of cervical cancer development. 2. Self-efficacy and outcome expectations to assess students’ motivation toward adhesion to cervical cancer prevention, through self-efficacy for vaccination, for getting Pap test, for using condoms and for limiting sexual partners. 3. Intentions toward cervical cancer prevention. | **Knowledge:** Both the video and SMS + video interventions significantly increased knowledge about cervical cancer risk factors. The video intervention group showed a mean increase in global knowledge from 3.44 to 4.19 (p < .0001, η2 = .60), while the SMS + video group improved from 3.41 to 4.30 (p < .0001, η2 = .55). The SMS + video intervention had a more pronounced effect on the knowledge of the risk of not getting a Pap test, with scores increasing from 3.97 to 4.50 (p = .005, η2 = .24).  **Self-efficacy and outcome expectations:** Outcome expectations improved significantly in both groups, but the increase in self-efficacy was not statistically significant.  **Intentions:** The SMS + video intervention significantly increased the intention to get a Pap test, with scores rising from 4.50 to 4.82 (p = .010, η2 = .223). The video-only intervention did not show a significant increase in intentions. The SMS + video group showed significantly higher post-intervention scores for the intention to get a Pap test compared to both the control and video-only groups.  **Conclusion**:  The study demonstrated that combining SMS with video intervention was more effective in increasing knowledge about cervical cancer prevention and the intention to get a Pap test than the video intervention alone. The SMS component provided an additional positive effect on knowledge and intentions, particularly for getting a Pap test.  **Limitations**: Convenience sample and self-selected participants, lack random assignment to intervention groups, Unable to track whether SMS messages were read, No long-term effect assessment |

Article 16: **Human Papillomavirus (HPV) Health Savings as an Alternative Solution: HPV Vaccination Behavior in Adolescents^38^**

| **Study** | **Sample Characteristics** | **Study Design** | **Intervention** | **Outcomes** | **Main Results** |
| --- | --- | --- | --- | --- | --- |
| **Title:** Human Papillomavirus (HPV) Health Savings as an Alternative Solution: HPV Vaccination Behavior in Adolescents  **Authors**: Wiwin Lismidiati, Ova Emilia, Widyawati Widyawati (2020)  **Purpose**: To measure the effects of reproductive health savings (Takespro) on HPV vaccine initiation and the quality of decision-making to get vaccinated, as measured by knowledge, attitudes, beliefs, and self-efficacy toward HPV vaccination. | **Region**: Yogyakarta, Indonesia  **Setting**: Junior high schools in the Special Region of Yogyakarta  **Participants:** Female junior high school students who had not received the HPV vaccination  **Sample Size**: 128 students (40 in the intervention group, 88 in the control group)  **Age/Grade:** Junior high school students  **Sex**: Female  **Race**: Not specified, majority (95.3%) were Moslem (Religion, I believe) | **Design**: Randomized control trail  **Recruitment**: Seventh- and eighth-grade students from selected schools with an Adolescent Reproductive Health Counseling Program.  **Incentive**: Not specified | **Duration**: Six months (October 2018 to April 2019)  **Theory**: Health Belief Model  **Program**:  Health Education: "Takespro HPV" intervention through videos and booklets  Savings Program: Reproductive health savings initiative to fund the first dose of HPV vaccination | 1. Knowledge of HPV and HPV vaccine 2. Attitude toward HPV vaccine 3. Belief toward HPV vaccine 4. Self-efficacy toward HPV vaccine 5. Reproductive Health Saving: it is regarding to enable the purchasing power of students and parents of the HPV vaccine to uptake HPV vaccination behavior. | **Knowledge of HPV and HPV vaccine:** The intervention group showed a significant improvement in HPV knowledge, with a mean score increase from -3 to 11, compared to the control group's increase from -9 to 7 (p = 0.016). The intervention group also demonstrated a significant increase in self-efficacy related to HPV vaccination, with a mean score improvement of 3.7 ± 7.9 compared to the control group's 0.6 ± 4.3 (*p* = 0.022).  **Self-efficacy:** Participants’ self-efficacy in the intervention group was significantly higher than the control group (*p* = .02). Meta-analysis: Intervention: n = 40, 3.7 ± 7.9; Control n = 88, 0.06 ± 4.3  **Attitudes and Beliefs:** Although there were improvements in attitudes (mean score of 3 ± 4.7 for the intervention group and -2 ± 3.5 for the control group) and beliefs (mean score of 1.8 ± 4.0 for the intervention group and 0.6 ± 3.4 for the control group), these differences were not statistically significant (*p* = 0.576 for attitudes, *p* = 0.110 for beliefs).  **Reproductive Health Savings:** The savings program encouraged students to save money for HPV vaccination, but the amount saved over six months was insufficient to cover the cost of vaccination. Students saved between IDR 63,000 and IDR 200,000, which was not enough to finance the initial dose of the HPV vaccine.  **Limitations**: Short savings period insufficient to cover the cost of HPV vaccination, Convenience sampling and self-selected participants, Limited generalizability due to small sample size, Incomplete data on parental consent and decision-making |

Article 17: **Knowledge of HPV in West Virginia High School Health Students and the Effects of an Educational Tool^33^**

| **Study** | **Sample Characteristics** | **Study Design** | **Intervention** | **Outcomes** | **Main Results** |
| --- | --- | --- | --- | --- | --- |
| **Title:** Knowledge of HPV in West Virginia High School Health Students and the Effects of an Educational Tool  **Authors**: Maria D. Merzouk, Pam Courtney, Sheli Garrett-Albaugh, Jabin Janoo, Gerald Hobbs, Michael Vernon (2011)  **Purpose**: To assess the knowledge of HPV in West Virginia high school students, create an educational DVD to be used as a supplement to health class, and evaluate whether the intervention increases student knowledge of HPV. | **Region**: West Virginia, United States  **Setting**: High school health classes in three West Virginia counties  **Participants:** High school students enrolled in health education classes  **Sample Size**: 626 students (372 in the HPV DVD intervention group, 254 in the control group)  **Age/Grad**e: Not specified, but the vast majority were high school freshmen  **Sex**: both male and female students  **Race**: Not Specified | **Design**: Prospective, randomized experimental design with analysis of questionnaire data  **Recruitment**: Eligible subjects were all high school students enrolled in the school’s health education classes. Participation was voluntary, with informed consent obtained from both students and their parents or guardians.  **Incentive**: Not Specified. | **Duration**: Conducted over two separate days during the STI curriculum in health class  **Theory**: Based on educational principles aimed at increasing awareness and understanding of HPV  **Program**:  Educational DVD: A 15-minute HPV educational DVD created by healthcare providers  Health Class: Regular health education curriculum covering STIs, including HPV | Knowledge of HPV consisting of 11 true/false questions to measure knowledge about HPV | **Knowledge of HPV:**  The control group showed a marginal increase in their average score from 70.56% on the pretest to 71.87% on the posttest (a 1% improvement). The intervention group, who watched the HPV DVD, showed a more significant increase in their average score from 74.14% on the pretest to 81.48% on the posttest (a 7% improvement). This improvement was statistically significant (p < .0001).  The intervention group scored higher on 9 out of the 11 questions in the posttest compared to the control group. Significant improvements were observed in the intervention group for questions related to HPV knowledge.  Understanding that most women with HPV do not have abnormal menstrual periods increased from 40.32% to 64.52% (p < .05).  Knowing that HPV infection is often detected by a Pap test increased from 80.65% to 93.82% (p < .05).  **Limitations**: Lack of standardization in health classes, No demographic data collected, Absenteeism leading to unmatched pre- and post-test data, No use of graphics in the educational material due to parental concerns |

Article 18: **Helping Children to Participate in Human Papillomavirus–Related Discussions: Mixed Methods Study of Multimedia Messages^39^**

| **Study** | **Sample Characteristics** | **Study Design** | **Intervention** | **Outcomes** | **Main Results** |
| --- | --- | --- | --- | --- | --- |
| **Title:** Helping Children to Participate in Human Papillomavirus–Related Discussions: Mixed Methods Study of Multimedia Messages  **Authors**: Aurora Occa, Hayley M. Stahl, Sarah Julien-Bell (2022)  **Purpose**: To assess the feasibility of using an evidence-based animated video and a web-based game to help children (aged 11-12 years) participate in discussions about their health, particularly regarding HPV vaccination, and to improve HPV-related outcomes. | **Region**: Italy  **Setting**: Large public school in northern Italy  **Participants:** Middle school children aged 11-12 years  **Sample Size**: 35 children (20 watched the animated video, 15 played the game)  **Age/Grad**e: 11-12 years, second year of middle school  **Sex**: Male and 57% female (20/35)  **Race**: Not specified, Italian children | **Design**: Randomized controlled trial  **Recruitment**: Teachers distributed leaflets and informed consent documents to parents of children enrolled in the second year of middle school.  **Incentive**: Not Specified | **Duration**: Focus groups conducted in January 2020  **Theory**: Theory of Planned Behavior (TPB), Social Cognitive Theory (SCT), and Gamification approach  **Program**:  Animated Video: "Salute e HPV (Health and HPV)"  Game: "Salute e HPV (Health and HPV)" web-based quiz game  **Assessment**: Pre- and post-intervention questionnaires measuring knowledge, attitudes, intention to talk, self-efficacy, subjective norms, and fear  **Data Analysis**: Qualitative analysis of focus group discussions using NVivo Analyses of covariance for experimental data Descriptive statistics and t-tests for comparing pre- and post-intervention scores | 1. Knowledge of HPV, 2. Attitudes toward HPV vaccine, 3. Intention to talk about the HPV vaccine, 4. Self-efficacy toward the HPV vaccine, 5. Subjective Norms of getting the HPV vaccine 6. Fear regarding the HPV vaccine   **Video**:  Both the animated video and the game were well-received, with high enjoyment scores (4.45 for the video and 4.60 for the game). Children found both formats engaging and appreciated the characters and presentation styles.  Children appreciated the bright and attractive colors and the clear, detailed explanations. The animated video’s use of a whiteboard to illustrate concepts and the game’s informative summaries were particularly praised.  **Suggestions for Improvement:**  Children suggested including more information on the side effects of the vaccine and symptoms of HPV. They expressed a desire for the materials to be more widely accessible online to share with parents and peers.  The health care professional in the video was liked for her caring attitude and clear explanations. The diversity and relatability of the young characters were positively noted, though some felt the characters appeared younger than their actual age. | **Knowledge of HPV**: Both the animated video and the game significantly increased children's knowledge about HPV. The mean knowledge score for the video increased from 3.34 to 3.90 (mean difference = 0.55, p < .001), while for the game, it increased from 3.34 to 3.79 (mean difference = 0.45, p < .001).  **Attitude, Intention, self-efficacy and subjective norms**:  The video significantly improved children's intention to discuss the HPV vaccine (mean difference = 0.60, p < .001), though it did not significantly change attitudes or subjective norms. The game also improved intention significantly (mean difference = 0.80, p = .01), but not attitudes.  The video improved self-efficacy (mean difference = 0.25, p < .001), but the game did not show a significant effect. Neither the video nor the game significantly changed subjective norms.  **Fear Reduction:** The video effectively reduced fears related to the HPV vaccine (mean difference = -0.25, p < .001), while the game did not show a significant change.  **Limitations**: Small sample size, conducted in only one school in northern Italy, lack of parental and physician feedback, translated scales not previously validated with an Italian audience |
